# Questing functions and structures of hypothetical proteins from *Campylobacter jejuni*: a computer-aided approach

**DOI:** 10.1042/BSR20193939

**Published:** 2020-06-09

**Authors:** Md. Amran Gazi, Sultan Mahmud, Shah Mohammad Fahim, Md. Rezaul Islam, Subhasish Das, Mustafa Mahfuz, Tahmeed Ahmed

**Affiliations:** 1 Nutrition and Clinical Services Division, International Centre for Diarrhoeal Disease Research, Bangladesh (icddr,b), Bangladesh; 2 Infectious Diseases Division, International Centre for Diarrhoeal Disease Research, Bangladesh (icddr,b), Bangladesh; 3 Neurosciences and Molecular Biosciences, International Max Planck Research School, Grisebachstraße 5, 37077 Göttingen, Germany; 4 Faculty of Medicine and Life Sciences, University of Tampere, Tampere, Finland

**Keywords:** Campylobacter, gastroenteritis, hypothetical protein, in silico, NCBI

## Abstract

*Campylobacter jejuni* (*C. jejuni*) is considered to be one of the most frequent causes of bacterial gastroenteritis globally, especially in young children. The genome of *C. jejuni* contains many proteins with unknown functions termed as hypothetical proteins (HPs). These proteins might have essential biological role to show the full spectrum of this bacterium. Hence, our study aimed to determine the functions of HPs, pertaining to the genome of *C. jejuni*. An *in-silico* work flow integrating various tools were performed for functional assignment, three-dimensional structure determination, domain architecture predictors, subcellular localization, physicochemical characterization, and protein–protein interactions (PPIs). Sequences of 267 HPs of *C. jejuni* were analyzed and successfully attributed the function of 49 HPs with higher confidence. Here, we found proteins with enzymatic activity, transporters, binding and regulatory proteins as well as proteins with biotechnological interest. Assessment of the performance of various tools used in this analysis revealed an accuracy of 95% using receiver operating characteristic (ROC) curve analysis. Functional and structural predictions and the results from ROC analyses provided the validity of *in-silico* tools used in the present study. The approach used for this analysis leads us to assign the function of unknown proteins and relate them with the functions that have already been described in previous literature.

## Introduction

*Campylobacter* is the genus that comprises a diverse group of non-spore forming rod-like or spiral-shaped Gram-negative bacteria [1]. In developing countries, infections with *Campylobacter* are common in children under 2 years of age and found to be associated with increased incidence of diarrheal diseases as well as mortality [1,2]. In industrialized nations, *Campylobacter* is the cause of diarrhea during early years of adulthood [3]. *Campylobacter* infections are mostly acquired through consumption of contaminated water and food in resource-poor environment [4]. Two of the species, *C. jejuni* and *C. coli*, are primarily known to be responsible for human campylobacteriosis [4]. Acute gastroenteritis and food poisoning can be induced by *C. jejuni* in infected patients. Usually, *C. jejuni* infection causes gastroenteritis without any complication but acute infection may results in abdominal cramps, fever or other ailments like Guillain–Barré syndrome or Miller Fisher syndrome [5]. Recent studies also showed an association of *Campylobacter* infections with malnutrition, a condition highly prevalent in developing countries [2].

Although whole genome sequence of *C. jejuni* NCTC has been published, a detailed catalog of prospective virulence is yet to be documented. Its complete genome contains a circular chromosome of 1641481 base pairs with GC content: 30.6%. Several studies since then suggest *C. jejuni* exhibits high genomic diversity across strains. A shotgun DNA microRNA approach revealed 63-kb long unique genomic DNA sequences in another *Campylobacter* strain, *C. jejuni* 81–176 when compared with fully sequenced *C. jejuni* NCTC 11168, implying genetic diversity between strains [6,7]. Overall, genome of *C. jejuni* strain 81–176 (total length 1.6 Mb) available in NCBI encodes 1658 proteins (GC%: 30.4) [7]. Among them 267 are yet to be experimentally determined, and are designated as hypothetical proteins (HPs). Similar to functionally annotated proteins, HP originates from an open reading frame (ORF), but lacks functional annotations [8]. Therefore, annotation of HPs of specific organism leads to the introduction of unique functions, and helps in listing auxiliary protein pathways [8].

Several contemporary bioinformatics tools, for instance, CDART, SMART, Pfam, INTERPROSCAN, MOTIF, SUPERFAMILY, and SVMProt have been well established to specify the functions of many bacterial HPs [9–11]. Besides, the exploration of protein–protein interaction (PPI) for instance, using STRING database [12], is crucial for comprehending the aspect of biological network. During cellular processes protein interactions play an essential role. Thus, an understanding of HP function can be reached by studying the PPIs [13]. Consequently, interaction of one protein and their function is proven to be dependent on the regulatory connection with other protein [54]. Three-dimensional modeling is also a great way to relate structural knowledge with the function of undetermined proteins [14]. Protein structure is generally more conserved than protein sequence [15]. Therefore, structural determination is considered to be a strong indicator of similar function in two or more proteins. Moreover, evolutionary distant proteins and its function can also be identified through structural information [15].

Functional prediction of HPs by using *in silico* approaches has been successfully applied for various bacteria and parasites [10,16,17]. In the present study, we have chosen *C. jejuni* as a template to explore the functions of HPs from its genome with a higher accuracy using well-optimized bioinformatics tools.

## Materials and methods

### Retrieval of genome data

Full genome of *C. jejuni* strain 81–176 was retrieved from NCBI (GCA_000015525.1, NC_008787.1). According to the repository this genome encodes 1658 proteins (http://www.ncbi.nlm.nih.gov/genome/), of which 267 are assigned as HPs. FASTA sequences of HPs were then retrieved for further analysis in the present study (accessed 27 February 2019).

### Functional analysis of HPs

In order to assign the function using the databases depicted in Supplementary Table S1, first we submitted proteins to five publicly available free tools (CDD-BLAST, HmmScan, SMART, Pfam, and SCANPROSITE) [18–22]. These databases can search for the conserved domains and subsequently help in the categorization of proteins. Analyses of HPs by five webtools revealed the distinct results. To find a composite result, different confidence levels were assigned on the basis of pooled results obtained from five webtools. For instance, if we observed same results from the five distinct tools, the composite score was 100 (percentage of confidence). For downstream analyses, we filtered 50 out of 267 HPs that displayed 60% or above confidence (Supplementary Table S2).

**Figure 1:**
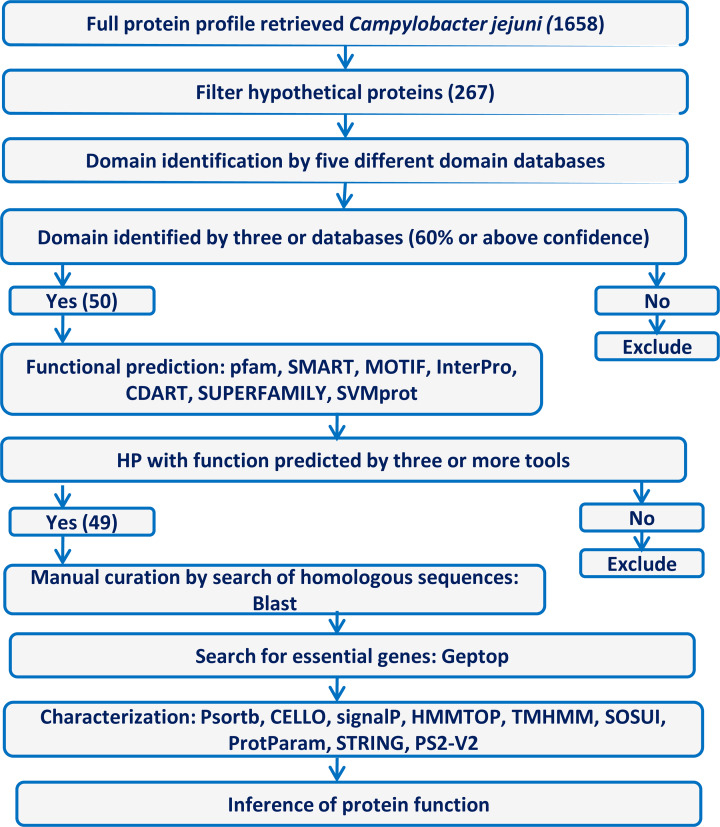
Flow chart showing the overall design of the study

Next, we performed functional assignment of these 50 selected HPs using different tools (Figure 1). SMART and CDART [23] facilitated to look for functions using the domain architecture and conserved domain database, respectively. To classify HPs into functional families based on similarity, we employed SUPERFAMILY [24], Pfam [21], and SVMProt [25]. Software such as InterPro and MOTIF search tool were also used to detect the motif in the proteins [26,27]. Default parameters were used for all these databases.

We further annotated HPs manually through searching for homologous proteins from related organisms. To do this, we used BLAST against the NCBI nonredundant (nr) database. If the two sequences were ≥90% identical, we considered it as homologues to each other. Query cover, score parameters and e-value of every hit are summarized in Supplementary Material S5.

Geptop 2.0 database was used to identify the essential genes among the HPs [28]. Default essentiality score cutoff of 0.24 was adopted. Geptop is the essential gene identification tool based on phylogeny and orthology. In the present study, a similarity search was also done against DrugBank 3.0 for all the targets [29].

### Prediction of physicochemical characteristics

Expasy’s ProtParam server was used for extinction coefficient, isoelectric point (pI), molecular mass, instability index, aliphatic index, and grand average of hydropathicity (GRAVY) prediction [30].

### Identification of subcellular localization

PSORTb [31] and CELLO [32] were applied to find the localization of HPs in the cell. PSORTb contains the information both from laboratory experimentations and *in silico* prediction. On the other hand, a support vector machine was used by CELLO database to generate the probable localization of protein in the cell. TMHMM [33], SOSUI [34], HMMTOP [35], and SignalP [36] were also applied to detect membrane protein and to verify the presence of cleavage sites for peptide.

### Functional protein association networks

We had employed STRING software [37] to predict interactive partners of HPs in this investigation. This database computes the network based on physical and functional associations. Highest score network proteins were selected for this analysis in order to accord the reliability of the PPIs.

### Determination of three-dimensional structures

Structure prediction of a protein from its sequences is a way that enables the identification of function. A template based online server PS2-v2 was used to predict the tertiary structure of the HPs in this study [38]. This server uses a template of known protein structures and then applied the approaches of multiple and pairwise alignments combining IMPALA, T-COFFEE, and PSI-BLAST.

### Performance assessment

A receiver operating characteristic (ROC) was implemented to confirm the accuracy of the predicted functions of HPs from *C. jejuni* genome. First, we selected 40 proteins randomly with known functions of *C. jejuni* (Supplementary Table S3). These proteins were predicted for the functions using the same databases that were used for the prediction of HPs. To classify the prediction, true positive (1) and true negative (0) were denoted as binary numerals. Six levels diagnostic efficacy was also evaluated where the integers ‘2’, ‘3’, ‘4’, and ‘5’ were used. A web-based calculator was applied to submit the classification data for ROC curve and is utilized to calculate the sensitivity, specificity, ROC area, and accuracy of the tools used to speculate the function of HPs [39].

## Results and discussion

### Analysis of HPs from *C. jejuni* genome

With the ongoing developments of DNA sequencing technologies called high throughput sequencing techniques has enabled a substantial number of bacterial genome sequencing. Annotation of the genes generally depends on sequence homology techniques [40]. However, a large number of genes have no assigned function. Therefore, only homology techniques cannot assign functions precisely and may lead to incorrect annotations [41]. Multiple tools should be used to avoid this problem to assign functions of HPs. Hence, the present study focused on the annotation of HPs from *C. jejuni* using assorted but effective bioinformatics tools.

**Figure 2:**
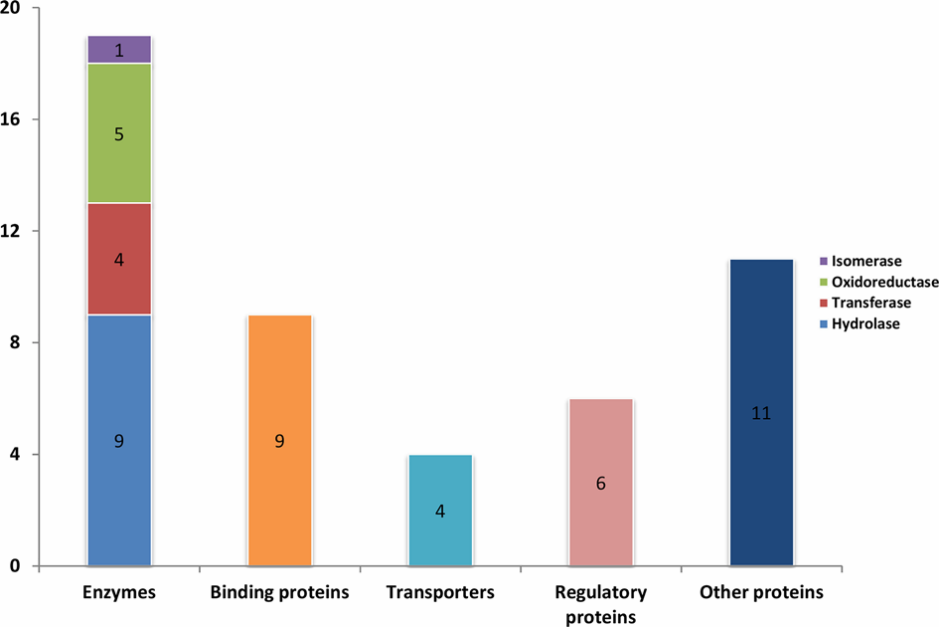
Functional classification of 49 HPs into various groups

First, functional domains were identified from the sequences of all the 267 HPs using SCANPROSITE, SMART, Pfam, CDD-BLAST, and HmmScan. Specific domains could be identified using one, two, three, four, or five of the above-stated tools and therefore, different confidence levels were assigned (e.g., 20, 40, 60, 80, and 100%). In our previous studies, published elsewhere, we only considered the proteins with 100% confidence [10,42]. However, in the current study, HPs having 60% or above confidence level have been considered to gain the greater coverage. The analyses revealed 50 such proteins which were used for downstream analyses. For rest of the HPs (n=217), domains were recognized from one or two of the mentioned tools. Further studies are needed to find the exact function for these proteins. Supplementary Table S2 summarized protein lists with domain. The final pool of 50 proteins was examined employing CDD-BLAST, Pfam, SMART, MOTIF, InterPro, CDART, SUPERFAMILY, and SVMProt. Functional annotation was considered to be high for proteins that manifested same function from equal or more than three tools (Supplementary Table S4). Thus, we inferred 49 such proteins with high confidence (Table 1) and classified them as highly confident proteins (Hconf), where 11 contain homologous sequences without product function reported (Supplementary Table S5). Analyses of sequence were then accumulated and Hconf proteins were grouped into different functional categories. Functional classes of proteins consists of regulatory proteins, transporters, binding proteins, enzymes, proteins with biotechnological interest, and proteins with other functions (Figure 2). The categorization was selected based on the literature search and gene ontology. Enzyme classes were determined from enzyme data bank of Expasy (https://enzyme.expasy.org/cgi-bin/enzyme/enzyme-search-cl?2).

**Table 1:** HPs functionally annotated from *C. jejuni*

No.	Protein IDs	Protein function
1	WP_002868767.1	Curli production assembly, transport component CsgG
2	WP_002854524.1	Chemotaxis phosphatase CheX
3	WP_009882162.1	SprA-related family
4	WP_010790856.1	Pyridoxamine 5′-phosphate oxidase
5	WP_009882239.1	Hemagglutination activity domain
6	WP_002854991.1	FxsA cytoplasmic membrane protein, FxsA
7	WP_002855029.1	DNA replication regulator, HobA
8	WP_002868905.1	GDSL-like lipase
9	WP_002869356.1	Divergent polysaccharide deacetylase
10	WP_002856929.1	C4-type zinc ribbon domain
11	WP_002869028.1	Esterase-like activity of phytase
12	WP_011812736.1	Domain of unknown function DUF234
13	WP_002868809.1	Ankyrin repeats, Ank_2
14	WP_002869368.1	Type-1V conjugative transfer system mating pair stabilization, TraN
15	WP_009882583.1	NLPC_P60 stabilizing domain
16	WP_002853389.1	Jag, N-terminal domain superfamily
17	WP_009882608.1	Adhesin from *Campylobacter*
18	WP_002856369.1	Putative β-lactamase-inhibitor-like
19	WP_079254190.1	β-1,4-N-acetylgalactosaminyltransferase (CgtA)
20	WP_002856180.1	Heavy-metal-associated domain
21	WP_002831611.1	Transcription factor zinc-finger
22	WP_002790076.1	Methyl-accepting chemotaxis protein (MCP) signaling domain
23	WP_002853792.1	Plasminogen-binding protein pgbA N-terminal
24	WP_002869072.1	Putative S-adenosyl-l-methionine-dependent methyltransferase
25	WP_002869097.1	MaoC-like dehydratase domain
26	WP_002869326.1	Metallo-carboxypeptidase
27	WP_002869139.1	Pyruvate phosphate dikinase, PEP
28	WP_002869195.1	Anti-sigma-28 factor
29	WP_002856630.1	PD-(D/E)XK nuclease superfamily
30	WP_002855458.1	MgtE intracellular N domain
31	WP_002797496.1	Flagellar FliJ protein
32	WP_024088174.1	Nitrate reductase chaperone
33	WP_009883030.1	ATPase, AAA-type, core
34	WP_002824979.1	Putative NADH-ubiquinone oxidoreductase chain E
35	WP_002869225.1	DMSO reductase anchor subunit (DmsC)
36	WP_002856602.1	Putative β-lactamase-inhibitor-like
37	WP_002868888.1	Tetratricopeptide repeat, TPR_2
38	WP_002868880.1	ABC-type transport auxiliary lipoprotein component
39	WP_009883121.1	Flagellar FLiS export co-chaperone
40	WP_002860117.1	Menaquinone biosynthesis
41	WP_002779704.1	T-antigen specific domain
42	WP_011187233.1	Toprim domain
43	WP_011187235.1	AAA domain, AAA_25
44	WP_002809111.1	TrbM superfamily
45	WP_011117548.1	Bacterial virulence protein VirB8
46	WP_011117549.1	Conjugal transfer protein
47	WP_011117575.1	Type IV secretion system proteins,T4SS
48	WP_011799393.1	TrbM superfamily
49	WP_011117588.1	mRNA interferase PemK-like

Moreover, essential genes were predicted using Geptop, a database that accommodates already sequenced bacterial genomes. These genes are fundamental for survival of an organism and perform essential activities of the cell [43]. Identification of essential genes is an important stride toward gaining better insight into the evolution [44]. Time-absorbing and challenging experiential procedures like transposon mutagenesis, RNA interference, and single-gene knockouts were used to identify essential genes [28]. However, *in-silico* approaches offer an alternative for predicting essential genes. In the current study, it was possible to identify 32 essential proteins by using Geptop database (Supplementary Table S6). Besides, from the selected Hconf proteins, only one protein was found to be exhibited similarity with approved drugs. The test was done through protein BLAST against DrugBank. Protein WP_002868809.1 showed the similarity with fostamatinib that could act as inhibitors. DrugBank contains 6816 FDA-approved and experimental drugs, 169 drug enzymes/carriers, and 4326 drug targets.

Finally, ROC curve was calculated to identify the reliability of the tools used to predict the function. Average accuracy was found to be 95% for the used pipeline and area under the curve (AUC) was 0.97 (Table 2). It is recommended to use the AUC to summarize the overall accuracy of the tools in the diagnosis [45]. The AUC value ranges from 0 to 1, and the value greater than 0.7 is considered acceptable [45]. The ROC analyses results provided the high reliability of *in-silico* tools used in our study (Table 2). However, predicting the functions of the ‘function-known’ proteins and obtaining very high accuracy does not mean the prediction on ‘function-unknown’ proteins would reproduce the same level of accuracy.

**Table 2:** ROC results of various tools used in the present study

No.	Software	Accuracy (%)	Sensitivity (%)	Specificity (%)	ROC area
1	PFAM	95%	94.7%	100%	0.97
2	SMART	95%	94.9%	100%	0.97
3	MOTIF	95%	94.9%	100%	0.97
4	INTERPROSCAN	95%	94.9%	100%	0.97
5	CDART	97.5%	97.4%	100%	0.99
6	SUPERFAMILY	95%	94.1%	100%	0.97
7	SVMprot	90%	88.9%	100%	0.94
8	Average	**95%**	**94.3%**	**100%**	**0.97**

### Enzymes

We found five oxidoreductases among these HPs of *C. jejuni*. These enzymes play key role in the pathogenesis. WP_002824979.1 is an NADH-quinone oxidoreductase, an enzyme that involves in regulating the expression of virulence factors, electron transport, and sodium translocation [46]. This putative domain commonly found in Epsilonproteobacteria, chiefly in *Helicobacter pylori* (*H. pylori*) [47]. Protein WP_002869225.1 is dimethyl sulfoxide reductase that acts as the terminal electron transfer enzyme in *Escherichia coli* (*E. coli*). This enzyme and the reaction it catalyzes could prove helpful on the climate control frontier [48]. We also found four proteins as transferase those might involved in bacterial pathogenesis and virulence. Among them, protein WP_002854524.1 is responsible for modifying the bacterial character in the presence of repellents and nutrients, found in chemotaxis phosphatase CheX [49]. Hydrolases is the third class of enzymes where almost 50% proteins among all characterized enzymes represent this class. This class of proteins is generally membrane-bound involved in various virulence factors associated with metal ion binding, transmembrane transport, cell wall degradation. We have found WP_002856630.1 that represents endonuclease-like domain involved in DNA repair and replication [50]. WP_009883030.1 and WP_011187235.1 exhibit AAA ATPases (ATPases associated with diverse cellular activities) which plays a number of role in the cell including protein proteolysis and disaggregation, cell-cycle regulation, organelle biogenesis, and intracellular transport [51]. In addition WP_011187233.1 protein is a toprim (topoisomerase-primase) domain that is found in bacterial DnaG-type primases, involved in DNA strand breakage and rejoining [52].

### Binding

We have identified nine proteins as binding among the functionally annotated HPs. These can be further classified into RNA binding, DNA binding, protein binding, ion binding, and adhesion proteins. Binding of proteins is important in the propagation and survival of pathogens in the host [53]. For example, protein binding WP_002868888.1 is tetratricopeptide repeat (TPR) motifs, reported to be directly related to virulence-associated functions [54]. WP_002853792.1 is the N-terminal domain of the bacterial proteins (PgbA) that bind to host cell protein, plasminogen [55]. This activity was identified in *H. pylori* where it is thought to contribute to the virulence of this bacterium [55]. WP_011117588.1 is mRNA interferase PemK-like domain, a growth inhibitor in *E. coli*. It is responsible for mediating cell death through inhibiting protein synthesis [56]. Besides, WP_009882239.1 is a hemagglutination activity domain found in a number of large, repetitive proteins of bacteria. Filamentous hemagglutinin (FHA) is a secreted and surface-exposed protein that acts as main virulence attachment factor in childhood whooping cough caused by *Bordetella pertussis* [57]. WP_002868809.1 is found to be ankyrin repeat (ANK), a typical PPI motif in nature. A large number of bacterial pathogens mimic or manipulate various host functions through delivering ANK-containing proteins into eukaryotic cells [58]. Finally, WP_009882608.1 is adhesion protein called surface-exposed lipoprotein JlpA, an early critical step in the pathogenesis of *C. jejuni* disease [59]. This HP might provide new approach for the rational design of small molecule inhibitors against *C. jejuni* targeting JlpA efficiently [59].

### Regulatory

There are six HPs found to be involved in regulatory and cellular mechanisms, and are essential for the pathogenesis of *C. jejuni*, hence can be treated as probable drug targets. WP_002869195.1 is found to be anti-sigma-28 factor that inhibits the activity of the sigma 28 transcription factor. This inhibition prevents the expression of genes from flagellar transcriptional class 3, which include genes for chemotaxis. Mechanism of action of anti-sigma factors has opened new door on the regulation of bacterial gene expression, as anti-sigma factors join another layer to transcriptional control via negative regulation. The bacteriophage T_4_ uses an anti-sigma factor in order to transcribe its own genes by sabotaging the *E. coli* RNA polymerase [60]. WP_002797496.1 is a membrane-associated protein that affects chemotactic events. FliJ is a component of the flagellar export and has a chaperone-like activity. Mutations in FliJ result in failure to respond to chemotactic stimuli [61]. Moreover, WP_011117549.1 is identified as conjugal transfer protein that bacteria utilize to export effector molecules during infection. For example, *H. pylori* use type IV machines to transport effectors to the extracellular environment or cell cytosol of mammals [62]. A DnaA binding protein (WP_002855029.1) HobA, identified that is an essential regulator of DNA replication in *H. pylori* [63]. WP_002790076.1 is methyl-accepting chemotaxis protein (MCP) that allows bacteria to sense the concentrations of molecules (nutrients/toxins) in the extracellular milieu so that they can smooth swim or fall accordingly [64].

### Transporters

Transporter proteins are involved various metabolic processes, are responsible for transportation of nutrients, and hence, essential for survival of the organism. Besides, they accelerate the movement of virulence factors and are directly involved in pathogenesis [65]. WP_002855458.1 is the magnesium transporter E (MgtE), found in eukaryotic proteins. Magnesium (Mg^2+^) is an essential element for growth and maintenance of living cells where MgtE transports magnesium across the cell membrane [66]. WP_002868880.1 is an ABC-type transport, responsible for outer membrane biosynthesis in bacteria that can be an excellent drug target [67]. WP_002856180.1 is heavy metal-associated (HMA) domain found in a number of detoxification proteins or in heavy metals transport. Proteins that are involved in transporting heavy metals in bacteria, plants, and mammals share similarities across the kingdoms in their structures and sequences. These proteins provide an important arena for research, some being involved in bacterial resistance to toxic metals, while others are responsible for acquired human diseases, such as Wilson’s and Menke’s diseases [68]. WP_011117548.1 is the bacterial virulence protein VirB8 that is thought to be a constituent of DNA transporter. In addition, VirB8 is a potential drug target that targets its PPIs. X-ray structure has enabled a detailed structure–function analysis of VirB8, which identified VirB8 interaction with VirB4 and VirB10 [69]. Our results also go in line with this as we observed VirB8 has strong interaction with VirB10.

### Potential proteins with biotechnological applications

We identified few proteins that can have biotechnological applications based on their functional process. For instance, WP_010790856.1 is pyridoxamine 5′-phosphate oxidase (pdxH), an enzyme involved in the *de novo* synthesis of pyridoxal phosphate and pyridoxine (vitamin B6). Moreover, PdxH is evolutionarily related to phzD (also known as phzG), one of the enzymes in the phenazine biosynthesis protein pathway [70]. Only known source of phenazines are bacteria in nature. This is used as drug and also acts as biocontrol agents to inhibit plant pests. For example, the phenazine pyocyanin contributes to its potential to colonize the lungs of cystic fibrosis patients [71]. Similarly, phenazine-1-carboxylic acid, produced by a number of *Pseudomonas*, increases survival in soil and has been shown to be important for the biological control of certain strains [72]. The protein WP_002869072.1 was predicted to be S-adenosyl-L-methionine-dependent methyltransferase (SAM-MTase). Methyltransferases transfer a methyl group from a donor to an acceptor during methylation of biopolymers [73]. SAM-MT was used in the pharmaceutical industry as catechol, first as an antimicrobial and anticancer agent [73,74].

Protein WP_024088174.1 is the nitrate reductase that produces nitrite from nitrate. Nitrate is the primary source of nitrogen in fertilized soils and the reaction is critical for the production of protein in crop plants. Nitrate reductase enzyme activity can also be used as a biochemical tool for predicting grain protein production and subsequent grain yield. For example, it promotes amino acid content in tea leaves [75]. It is also reported that tea plants sprayed with various micronutrients (like Zn, Mn, and B) along with Mo enhanced the amino acid production of tea and the crop yield [75]. WP_002869028.1 is a phytase-like domain that catalyzes the hydrolysis of phytic acid. Phytic acid is organic form of phosphorus and indigestible found in grains and oil seeds. Phytase is produced by bacteria found in the gut of ruminant animals which are able to make phosphorus from phytic acid [76]. But, non-ruminants like human cannot make phytase. Research in the field of animal nutrition has put the idea of supplementing feed with phytase to make sure the availability of phytate-bound nutrients like phosphorus, calcium, carbohydrates, proteins, and other minerals [77].

Peptidase, an enzyme that is used as the ingredients of detergents, foods, and pharmaceuticals [78]. In this study, WP_009882583.1 was found to be cysteine peptidase that hydrolyzes a peptide bond utilizing the thiol group of cysteine as nucleophile. These peptidases are often confined to acidic environments and active at acidic pH such as the plant vacuole or animal lysosome. WP_002868905.1 is GDSL esterases and lipases are hydrolytic enzymes with broad substrate specificity. They have potential for use in the synthesis and hydrolysis ester compounds of biochemical, food, pharmaceutical, and other biological interests [79].

### Other proteins

WP_002856369.1 and WP_002856602.1 was found to be β-lactamase-inhibitor, a group of enzymes responsible for bacterial resistance to β-lactam antibiotics [80]. WP_009883121.1 s ass fla agellar FLiS export co-chaperone. Previously, various FliS-associated proteins in *H. pylori* were identified by a yeast two-hybrid study, but the implications are unknown [81]. Chaperones are usually involved in various important processes such as protein degradation, folding, and polypeptide translocation [81].

At last, WP_002860117.1 protein family includes two enzymes involved in menaquinone (vitamin K2) biosynthesis. In prokaryotes, vitamin K2 serves as the sole quinone molecule in electron shuffling systems while menaquinone pathway is absent from humans [82]. Therefore, novel antibacterial agents are possible to develop by targeting the bacterial enzymes responsible for menaquinone biosynthesis. It has been reported that inhibition of menaquinone showed significant growth inhibition against multidrug-resistant *Mycobacterium* and other Gram-positive bacteria as well as effective in killing Gram-negative bacteria [83].

### Prediction of primary properties and protein localization

Sequences of amino acids of 49 HPs were analyzed to evaluate their primary properties, and their localization (Supplementary Table S7). But, we paid attention to some proteins that showed functions important for the survival of *Campylobacter* and might have biotechnological interest. The proteins WP_024088174.1, WP_002869072.1, WP_010790856.1, WP_002868905.1, WP_002869028.1, WP_009882583.1 all had molecular weight (MW) values between 15792.47 and 52423.83. These proteins are referred to be biotechnologically important in the present study. Some proteins, essential for pathogenesis of *Campylobacter* have MW ranged from 8773.25 to 39113.6. The pI is the pH where protein carries no net electrical charge. For the list of mentioned proteins, it ranged from 5.03 to 9.63.

The aliphatic index indicates the protein thermostability [84]. Protein WP_002856369.1, associated with β-lactamase inhibition showed the highest values of 133.14. The GRAVY of protein indicates its hydrophobicity or the interaction with water [85]. In WP_002869028.1, WP_009882583.1, and WP_024088174.1, the scores are among −0.744, −0.439, and −0.393. Moreover, the instability index offers an assumption of the stability of protein *in vitro*. We used cut-off values >40 and <40 to discriminate between stable and unstable proteins, respectively. From our listed proteins, WP_024088174.1 and WP_002868880.1 were considered to be stable.

Localization plays an essential role in determining function of unknown proteins [11]. Protein WP_002868905.1 and WP_009882583.1 is located in outer membrane whereas other proteins of interest were predicted to be in the cytoplasm.

### PPI network

Function of a completely unknown protein can be identified based on the evidence of their interactions with the known proteins of a particular organism [11]. For example, PPI map and *in-vitro* proteome-wide interaction screens were applied to successfully assign the function of 50 unknown proteins for *Streptococcus pneumoniae* [86]. In our study protein WP_010790856.1, an oxidase (pdxH) showed a strong interaction with the Pyridoxine 5′-phosphate synthase that involved in vitamin B6 synthesis. WP_024088174.1 is interacted with formate dehydrogenase, an oxidoreductase that oxidizes formate to form carbon dioxide. WP_002868880.1 was found to be interacted with ABC transporter that functions to maintain the asymmetry of the outer membrane. All these predictions of functional partners have strengthened our findings of function predicted by using functional prediction tools (Supplementary Table S8).

### Three-dimensional structures

Structural genomics has become a robust way to determine the novel structures of proteins, especially via X-ray crystallography [87]. Determination of unannotated protein structures can often help us to discover unexpected family relationships, hence giving the idea of their probable functions. Proteins unrelated to existing PDB entries may represent new functions. In this case, structures homologous to other organisms have manifested as surrogates in drug discovery. For example, Nolatrexed, an anticancer drug was discovered using the structure of *E. coli* thymidylate synthase (46% sequence identity with human homolog) [87]. Kinase inhibitors to kill the *Plasmodium falciparum* were identified using structures of protein kinases from *Cryptosporidium* and *Toxoplasma* (61 and 74% sequence identity, respectively) [88].

In our study, PS2-v2 online server was used to model the three-dimensional structures of the Hconf proteins for *Campylobacter*. Among the 49 Hconf proteins, 24 proteins revealed same domain as function prediction tools used in the present study. In contrast, nine proteins showed discrepant results and no suitable templates were found for 16 proteins (Supplementary Table S9). Identity of model ranged from 54.5 to 91.6% and was constructed from closely related *Campylobacter* genus bacteria belonging to the *H. pylori, E. coli, Bacillus*, and *Clostridium*.

Based on the resolution and identity, two best models were WP_002797496.1 and WP_002854991.1, which were annotated as Flagellar FliJ protein and FxsA cytoplasmic membrane protein, respectively. The structure obtained for FliJ protein was determined by X-ray crystallography earlier and refined with diffraction data to 1.8-Å resolutions, which was solved by an ortholog isolated from *Saccharomyces cerevisiae* (PDB 2efrA). FxsA was determined by electron microscopy and refined with diffraction data to 4-Å resolutions and solved by an ortholog isolated from *Torpedo marmorata* (PDB 1oedB). Both these proteins showed the same function as predicted by other function prediction tools. Proteins with shared sequence typically display similar functions in this way.

## Conclusions

Protein function identification of a pathogen is an essential step to understand its cellular and molecular processes. In the present study, we used a computer-aided approach to assign the function of HPs from *C. jejuni*. We predicted the function to 49 HPs with a higher confidence. In addition, localization of protein and primary structure prediction were useful in supporting the specific characteristics of annotated proteins. Proteins were further explored for PPI and their tertiary structures. We have identified proteins with important functions including enzymes, transporters, binding and regulatory proteins as well as proteins with biotechnological interest. To summarize, our comprehensive analysis produces a better understanding of *C. jejuni* genome related HPs that would help to find novel therapeutic interventions and targets. Moreover, we have obtained an excellent result using the pipeline used in the present study and the method can be used to annotate the function of unknown proteins.

However, biochemical and clinical investigations are required to confirm the function of predicted proteins. Several studies have been conducted previously using the cumulative *in-silico* and *in-vitro/in-vivo* approach to investigate the function of unknown proteins. For instance, *in silico* approaches were used to predict the biological function of some of the unknown *Mycobacterium* proteins. The chosen proteins posses the α/β- hydrolase topological fold, characteristic of lipases/esterases which were further validated by wet lab experiments [89]. Combination of *in-silico and in-vitro/in-vivo* assays were also used to characterize the function of HPs from several other organisms [90–93]. Moreover, *in-silico* structure prediction methods were applied for drug discovery in the absence of x-ray structure of the target protein and again confirmed by *in-vitro* assays. Nonetheless, functional prediction merely on *in silico* methods requires careful integration of several computational tools into a single streamlined process. We hope that the information of HPs in the present study will be innovative for further *in-vitro/in-vivo* analysis on *C. jejuni*.

## Supplementary Material

Supplementary Tables S1-S9

## Abbreviations

ABC: ATP-binding cassette
ANK: ankyrin repeat
AUC: area under the curve
FDA: food and drug administration
GDSL: motif consensus amino acid sequence of Gly, Asp, Ser, and Leu around the active site Ser
GRAVY: grand average of hydropathicity
Hconf: highly confident protein
HMA: heavy-metal-associated
HP: hypothetical protein
MCP: methyl-accepting chemotaxis protein
MgtE: magnesium transporter E
ORF: open reading frame
pdxH: pyridoxamine 5′-phosphate oxidase
pI: isoelectric point
PPI: protein–protein interaction
ROC: receiver operating characteristic
SAM-MT: S-adenosyl-L-methionine-dependent methyltransferase
TPR: tetratricopeptide repeat
