# Supplementary material for: Questing functions and structures of hypothetical proteins from Campylobacter jejuni: a computer-aided approach
Source: Biosci Rep. 2020 Jun 9;40(6):BSR20193939. doi: 10.1042/BSR20193939 (PMC7284324; doi:10.1042/BSR20193939)
Supplement: Supplementary Tables S1-S9 [file BSR-2019-3939_supp.pdf]

S1 Table. List of bioinformatics tools and databases

Analyse	Bioinformatics tool	Version	URL
**Functional analysis and conserved domain**	Pfam	31.0	https://pfam.xfam.org/
	SMART	8.0	http://smart.embl-heidelberg.de/
	MOTIF	*	https://www.genome.jp/tools/motif/
	InterPro	66.0	https://www.ebi.ac.uk/interpro/
	CDART	*	https://www.ncbi.nlm.nih.gov/Structure/lexington/lexington.cgi
	SUPERFAMILY	1.75	http://supfam.org/SUPERFAMILY/index.html
	SVMProt	*	http://bidd2.nus.edu.sg/cgi-bin/svmprot/svmprot.cgi
	CDD-Blast	3.16	https://www.ncbi.nlm.nih.gov/Structure/cdd/wrpsb.cgi
	HmmScan	3.2.1	https://www.ebi.ac.uk/Tools/hmmer/search/hmmscan
	Scanprosite	*	https://prosite.expasy.org/scanprosite/
	Geptop	2.0	http://cefg.uestc.cn/geptop/
**Sub-cellular localization of the protein**	PSORTdb	3.0	http://db.psort.org/
	CELLO	2.5	http://cello.life.nctu.edu.tw/
	SignalP	5.0	http://www.cbs.dtu.dk/services/SignalP/
	HMMTOP	*	http://www.enzim.hu/hmmtop/
	TMHMM	2.0	http://www.cbs.dtu.dk/services/TMHMM/
	SOSUI	*	http://harrier.nagahama-i-bio.ac.jp/sosui/sosui_submit.html
**Physical-chemical characterization**	ProtParam	*	https://web.expasy.org/protparam/
**Protein-protein interaction network**	STRING	10.5	https://string-db.org/
**Structure prediction**	PS2-V2	3.0	http://ps2.life.nctu.edu.tw/
**Performance assessment**	ROC analysis calculator	*	http://www.rad.jhmi.edu/jeng/javarad/roc/JROCFITi.html

*Information not available

Table S2. Scores of conserved domain search for 267 HPs of *C. jejuni* strain 81-176 using CDD-Blast, Pfam, HmmScan, SMART and Scanprosite tools.

No.	Protein ID	CDD Blast	Pfam	HmmScan	SMART	Scanprosite	Percentage (%)
1	WP_002855595.1	1	0	0	0	0	20
2	WP_002868969.1	0	0	0	0	0	0
3	WP_002866317.1	0	0	0	0	0	0
4	WP_002824650.1	0	0	0	0	0	0
5	WP_009881605.1	0	0	0	0	0	0
6	WP_072238758.1	0	0	0	0	1	20
7	WP_024088096.1	0	0	0	0	0	0
8	WP_002857726.1	0	0	0	0	0	0
9	WP_002851763.1	0	0	0	0	0	0
10	WP_002857765.1	1	0	0	0	1	40
11	WP_002857751.1	0	0	0	0	0	0
12	WP_002868767.1	1	1	1	0	0	60
13	WP_009881781.1	0	0	0	0	0	0
14	WP_002868941.1	0	0	0	0	0	0
15	WP_002857300.1	0	0	0	0	0	0
16	WP_002851715.1	0	0	0	0	0	0
17	WP_002854663.1	0	0	0	0	0	0
18	WP_002851904.1	0	0	0	0	0	0
19	WP_002857297.1	0	0	0	0	0	0
20	WP_002870694.1	0	0	0	0	0	0
21	WP_011812694.1	0	0	0	0	1	20
22	WP_002869065.1	1	0	0	0	0	20
23	WP_002867117.1	0	0	0	0	0	0
24	WP_002854172.1	0	0	0	0	0	0
25	WP_002882716.1	0	0	0	0	0	0
26	WP_002854628.1	0	0	0	0	0	0
27	WP_079254179.1	0	0	0	0	0	0
28	WP_002851686.1	1	0	0	0	0	20
29	WP_002868751.1	0	0	0	0	0	0
30	WP_002859434.1	0	0	0	0	0	0
31	WP_002854718.1	0	0	0	0	0	0
32	WP_002854524.1	1	1	1	1	0	80
33	WP_002868919.1	0	0	0	0	0	0
34	WP_002857293.1	0	0	0	0	0	0
35	WP_002857540.1	0	0	0	0	0	0
36	WP_002854351.1	1	0	0	0	1	40

**37**	WP_011812706.1	0	0	0	0	1	20
**38**	WP_009882097.1	0	0	0	0	0	0
**39**	WP_002859472.1	0	0	0	0	0	0
**40**	WP_011812708.1	0	0	0	0	0	0
**41**	WP_002857599.1	0	0	0	0	1	20
**42**	WP_009882129.1	1	0	0	0	0	20
**43**	WP_009882150.1	0	0	0	0	1	20
**44**	WP_002869238.1	0	0	0	0	0	0
**45**	WP_009882162.1	1	1	1	1	0	80
**46**	WP_002859498.1	0	0	0	0	0	0
**47**	WP_002857569.1	0	0	0	0	0	0
**48**	WP_002869241.1	0	0	0	0	0	0
**49**	WP_002869242.1	0	0	0	0	0	0
**50**	WP_002869268.1	0	0	0	0	0	0
**51**	WP_010790856.1	1	1	1	1	0	80
**52**	WP_002859017.1	0	0	0	0	0	0
**53**	WP_002868796.1	0	0	0	0	0	0
**54**	WP_002868795.1	0	0	0	0	0	0
**55**	WP_002859022.1	0	0	0	0	0	0
**56**	WP_002868785.1	0	0	0	0	0	0
**57**	WP_002868784.1	1	0	0	0	1	40
**58**	WP_009882239.1	1	1	1	1	0	80
**59**	WP_002869315.1	1	0	0	0	0	20
**60**	WP_002856797.1	0	0	0	0	0	0
**61**	WP_002855217.1	0	0	0	0	0	0
**62**	WP_002854991.1	0	1	1	1	0	60
**63**	WP_002776186.1	0	0	0	0	0	0
**64**	WP_010790923.1	0	0	0	0	0	0
**65**	WP_002869301.1	0	0	0	0	0	0
**66**	WP_011812719.1	1	0	0	0	0	20
**67**	WP_011812720.1	0	0	0	0	0	0
**68**	WP_002854816.1	0	0	0	0	0	0
**69**	WP_002869110.1	0	0	0	0	0	0
**70**	WP_002855063.1	0	0	0	0	0	0
**71**	WP_002855029.1	1	1	1	1	0	80
**72**	WP_024088100.1	1	0	0	0	0	20
**73**	WP_079005306.1	0	0	0	0	1	20
**74**	WP_002868898.1	0	0	0	0	0	0
**75**	WP_002868905.1	1	1	1	1	0	80
**76**	WP_002869362.1	0	0	0	0	0	0

**77**	WP_002869356.1	1	1	1	1	0	80
**78**	WP_002855032.1	0	0	0	0	0	0
**79**	WP_074468912.1	0	0	0	0	0	0
**80**	WP_002852327.1	0	0	0	0	0	0
**81**	WP_002854947.1	0	0	0	0	0	0
**82**	WP_002852223.1	0	0	0	0	0	0
**83**	WP_002852050.1	0	0	0	0	0	0
**84**	WP_002858514.1	1	0	0	0	0	20
**85**	WP_002856929.1	1	1	1	1	0	80
**86**	WP_002825403.1	0	0	0	0	0	0
**87**	WP_002869028.1	1	1	1	1	0	80
**88**	WP_002869375.1	0	0	0	0	0	0
**89**	WP_009882410.1	1	0	0	0	0	20
**90**	WP_009882413.1	1	0	0	0	0	20
**91**	WP_002869370.1	0	0	0	0	1	20
**92**	WP_009882434.1	1	0	0	0	0	20
**93**	WP_002852615.1	0	0	0	0	0	0
**94**	WP_002856776.1	0	0	0	0	0	0
**95**	WP_011812736.1	1	1	1	1	0	80
**96**	WP_009882443.1	0	0	0	0	0	0
**97**	WP_002867958.1	0	0	0	0	0	0
**98**	WP_009882473.1	0	0	0	0	0	0
**99**	WP_002856908.1	0	0	0	0	0	0
**100**	WP_002868809.1	1	1	1	1	1	100
**101**	WP_002868812.1	0	0	0	0	0	0
**102**	WP_002856855.1	0	0	0	0	0	0
**103**	WP_002868815.1	1	0	0	0	0	20
**104**	WP_011812741.1	0	0	0	0	0	0
**105**	WP_002852485.1	1	0	0	0	0	20
**106**	WP_002860516.1	1	0	0	0	0	20
**107**	WP_002858755.1	0	0	0	0	0	0
**108**	WP_002857162.1	0	0	0	0	0	0
**109**	WP_002856886.1	0	0	0	0	0	0
**110**	WP_011812746.1	0	0	0	0	0	0
**111**	WP_002869164.1	0	0	0	0	0	0
**112**	WP_002857165.1	0	0	0	0	0	0
**113**	WP_002856266.1	0	0	0	0	1	20
**114**	WP_002911887.1	0	0	0	0	0	0
**115**	WP_002856425.1	0	0	0	0	0	0
**116**	WP_011812749.1	0	0	0	0	0	0

**117**	WP_002869368.1	1	1	1	1	0	80
**118**	WP_002853267.1	0	0	0	0	0	0
**119**	WP_002868960.1	0	0	0	0	0	0
**120**	WP_009882583.1	1	1	1	1	1	100
**121**	WP_002853389.1	1	1	1	1	0	80
**122**	WP_002854139.1	1	0	0	0	0	20
**123**	WP_002855981.1	0	0	0	0	1	20
**124**	WP_009882608.1	1	1	1	1	1	100
**125**	WP_011812755.1	0	1	1	0	0	40
**126**	WP_009882621.1	0	0	0	0	0	0
**127**	WP_002856369.1	1	1	1	1	0	80
**128**	WP_024088204.1	0	0	0	0	0	0
**129**	WP_002869126.1	0	0	0	0	0	0
**130**	WP_002869124.1	1	0	0	0	1	40
**131**	WP_002853000.1	0	0	0	0	0	0
**132**	WP_002853832.1	1	0	0	0	0	20
**133**	WP_002869121.1	1	0	0	0	0	20
**134**	WP_002856015.1	1	0	0	0	0	20
**135**	WP_002855841.1	1	0	0	0	0	20
**136**	WP_002866103.1	0	0	0	0	1	20
**137**	WP_002869049.1	0	0	0	0	0	0
**138**	WP_002856019.1	0	0	0	0	0	0
**139**	WP_002852822.1	0	0	0	0	0	0
**140**	WP_079254190.1	1	1	1	1	0	80
**141**	WP_002856180.1	1	1	1	1	1	100
**142**	WP_002831611.1	1	1	1	1	0	80
**143**	WP_002822505.1	0	0	0	0	0	0
**144**	WP_002852900.1	1	0	0	0	0	20
**145**	WP_002790076.1	1	1	1	1	1	100
**146**	WP_002868861.1	0	0	0	0	0	0
**147**	WP_002853792.1	1	1	1	1	0	80
**148**	WP_002859287.1	0	0	0	0	0	0
**149**	WP_002868857.1	0	0	0	0	0	0
**150**	WP_002853180.1	0	0	0	0	0	0
**151**	WP_002869072.1	1	1	1	1	0	80
**152**	WP_002866237.1	0	0	0	0	0	0
**153**	WP_002869074.1	0	0	0	0	0	0
**154**	WP_002869076.1	0	0	0	0	0	0
**155**	WP_002869078.1	1	0	0	0	0	20
**156**	WP_002869097.1	1	1	1	1	0	80

**157**	WP_002854125.1	0	0	0	0	0	0
**158**	WP_002869326.1	1	1	1	1	0	80
**159**	WP_002855905.1	0	0	0	0	0	0
**160**	WP_002856036.1	0	0	0	0	0	0
**161**	WP_002859649.1	0	0	0	0	0	0
**162**	WP_002869147.1	1	0	0	0	0	20
**163**	WP_002869378.1	0	0	0	0	1	20
**164**	WP_002855633.1	0	0	0	0	0	0
**165**	WP_002862076.1	0	0	0	0	1	20
**166**	WP_002869139.1	1	1	1	1	0	80
**167**	WP_002860363.1	0	0	0	0	0	0
**168**	WP_002818956.1	1	0	0	0	0	20
**169**	WP_002867263.1	0	0	0	0	0	0
**170**	WP_004315270.1	0	0	0	0	1	20
**171**	WP_002855347.1	1	0	0	0	0	20
**172**	WP_002858858.1	0	0	0	0	0	0
**173**	WP_002869194.1	1	1	1	1	0	80
**174**	WP_002869195.1	0	1	1	1	0	60
**175**	WP_002855438.1	0	0	0	0	0	0
**176**	WP_002856630.1	1	1	1	1	0	80
**177**	WP_002869204.1	0	0	0	0	0	0
**178**	WP_002858872.1	0	0	0	0	0	0
**179**	WP_002855458.1	1	1	1	1	0	80
**180**	WP_002797496.1	1	1	1	1	0	80
**181**	WP_024088174.1	1	1	1	1	0	80
**182**	WP_009883030.1	1	1	1	1	0	80
**183**	WP_002922487.1	0	0	0	0	0	0
**184**	WP_002824979.1	1	1	1	1	0	80
**185**	WP_002869225.1	1	1	1	1	0	80
**186**	WP_002856158.1	0	0	0	0	0	0
**187**	WP_002934257.1	0	0	0	0	0	0
**188**	WP_002856602.1	1	1	1	1	0	80
**189**	WP_009883110.1	0	0	0	0	1	20
**190**	WP_002778908.1	0	0	0	0	0	0
**191**	WP_002868888.1	1	0	0	1	1	60
**192**	WP_002855727.1	0	0	0	0	0	0
**193**	WP_002868880.1	1	1	1	1	1	100
**194**	WP_009883121.1	1	1	1	1	0	80
**195**	WP_002868874.1	0	0	0	0	0	0
**196**	WP_002869278.1	0	0	0	0	0	0

**197**	WP_002860117.1	1	1	1	1	0	80
**198**	WP_079254198.1	0	0	0	0	0	0
**199**	WP_002882243.1	1	0	0	0	1	40
**200**	WP_002869298.1	0	0	0	0	0	0
**201**	WP_002853105.1	0	0	0	0	0	0
**202**	WP_002790440.1	1	0	0	0	0	20
**203**	WP_002790442.1	0	0	0	0	0	0
**204**	WP_002790713.1	0	0	0	0	0	0
**205**	WP_002779702.1	0	0	0	0	0	0
**206**	WP_002779703.1	0	0	0	0	0	0
**207**	WP_002779704.1	0	1	1	1	0	60
**208**	WP_002790730.1	1	0	0	0	0	20
**209**	WP_002804244.1	0	1	1	0	0	40
**210**	WP_011271766.1	0	0	0	0	0	0
**211**	WP_002779777.1	0	0	0	0	0	0
**212**	WP_002809140.1	0	0	0	0	0	0
**213**	WP_002826068.1	0	0	0	0	0	0
**214**	WP_002804272.1	0	0	0	0	0	0
**215**	WP_011187233.1	1	1	1	1	1	100
**216**	WP_011187234.1	1	0	0	0	1	40
**217**	WP_002844160.1	0	0	0	0	0	0
**218**	WP_011815226.1	0	0	0	0	0	0
**219**	WP_011187235.1	1	1	1	1	0	80
**220**	WP_002809051.1	1	0	0	0	0	20
**221**	WP_002809052.1	0	0	0	0	0	0
**222**	WP_002842869.1	0	0	0	0	0	0
**223**	WP_011187239.1	0	0	0	0	0	0
**224**	WP_032592775.1	0	0	0	0	1	20
**225**	WP_002809111.1	1	1	1	1	0	80
**226**	WP_002809110.1	0	0	0	0	0	0
**227**	WP_002809107.1	0	0	0	0	0	0
**228**	WP_002834241.1	0	0	0	0	0	0
**229**	WP_002779751.1	0	0	0	0	0	0
**230**	WP_002909884.1	1	0	0	0	0	20
**231**	WP_008976813.1	0	0	0	0	0	0
**232**	WP_002801797.1	0	0	0	0	0	0
**233**	WP_011117548.1	1	1	1	1	0	80
**234**	WP_011117549.1	1	1	1	1	0	80
**235**	WP_011117551.1	0	0	0	0	0	0
**236**	WP_002815556.1	0	0	0	0	0	0

**237**	WP_011117559.1	0	0	0	0	0	0
**238**	WP_011117563.1	0	0	0	0	0	0
**239**	WP_010398003.1	0	0	0	0	0	0
**240**	WP_024088118.1	1	0	0	0	0	20
**241**	WP_024088119.1	0	0	0	0	0	0
**242**	WP_011117567.1	0	0	0	0	0	0
**243**	WP_011799391.1	0	0	0	0	1	20
**244**	WP_011117569.1	0	0	0	0	0	0
**245**	WP_011117570.1	0	0	0	0	0	0
**246**	WP_011117573.1	0	0	0	0	0	0
**247**	WP_011117574.1	1	0	0	0	0	20
**248**	WP_011117575.1	1	1	1	0	0	60
**249**	WP_011799393.1	1	1	1	1	0	80
**250**	WP_011117576.1	0	0	0	0	0	0
**251**	WP_011117578.1	0	0	0	0	0	0
**252**	WP_011117579.1	0	0	0	0	0	0
**253**	WP_011117580.1	0	0	0	0	0	0
**254**	WP_011799395.1	0	0	0	0	0	0
**255**	WP_011117582.1	0	0	0	0	0	0
**256**	WP_011117583.1	0	0	0	0	0	0
**257**	WP_002815407.1	0	0	0	0	0	0
**258**	WP_004306057.1	0	0	0	0	0	0
**259**	WP_011117585.1	0	0	0	0	0	0
**260**	WP_011117586.1	0	0	0	0	0	0
**261**	WP_079254173.1	0	0	0	0	0	0
**262**	WP_011117587.1	0	0	0	0	0	0
**263**	WP_011117588.1	1	1	1	1	0	80
**264**	WP_011117589.1	0	0	0	0	1	20
**265**	WP_011799397.1	0	0	0	0	0	0
**266**	WP_011799398.1	0	0	0	0	0	0
**267**	WP_011117593.1	0	0	0	0	0	0

Note: 0 = 0%, 1 = 25%

Table S3. List of annotated functions of 40 proteins with known function from C. jejuni using Pfam, SMART, MOTIF, INTERPROSCAN, CDART, SUPERFAMILY and SVMprot for ROC analysis.

			**PFAM**		**SMART**		**MOTIF**		**INTERPROSCAN**		**CDART**		**SUPERFAMILY**		**SVMprot**	
No.	Protein ID	Protein Name_Known function	Prediction	Score	Prediction2	Score2	Prediction3	Score3	Prediction4	Score4	Prediction5	Score5	prediction6	Score6	prediction 7	Score7
**1**	WP_009881324.1	DNA polymerase III subunit beta	DNA polymerase III subunit beta	1 (5)	DNA polymerase III subunit beta	1 (5)	DNA polymerase III subunit beta	1 (5)	DNA polymerase III subunit beta	1 (5)	DNA polymerase III subunit beta	1 (5)	DNA polymerase III subunit beta	1 (5)	Zinc-binding, All DNA-binding	1 (4)
**2**	WP_009881354.1	glutamate synthase	glutamate synthase	1 (5)	glutamate synthase	1 (5)	glutamate synthase	1 (5)	glutamate synthase	1 (5)	glutamate synthase	1 (5)	glutamate synthase	1 (5)	Manganese-binding, Zinc-binding	1 (4)
**3**	WP_002855601.1	CTP synthase	CTP synthase	1 (5)	CTP synthase	1 (5)	CTP synthase	1 (5)	CTP synthase	1 (5)	CTP synthase	1 (5)	Nitrogenase iron protein-like, Class I glutamine amidotransferases (GAT)	1 (2)	Forming Carbon-Nitrogen Bonds, Transferases - Glycosyltransferases	1 (2)
**4**	WP_011812682.1	cytochrome c biogenesis protein	cytochrome c biogenesis protein	1 (5)	cytochrome c biogenesis protein	1 (5)	cytochrome c biogenesis protein	1 (5)	cytochrome c biogenesis protein	1 (5)	cytochrome c biogenesis protein	1 (5)	No result	0 (2)	All lipid-binding proteins, Transferases - Glycosyltransferases	1 (2)
**5**	WP_009881534.1	transglycosylase	transglycosylase	1 (5)	transglycosylase	1 (5)	transglycosylase	1 (5)	transglycosylase	1 (5)	transglycosylase	1 (5)	transglycosylase	1 (5)	All DNA-binding, Magnesium-binding	1 (3)
**6**	WP_009881539.1	Bcr/CflA family efflux MFS transporter	Bcr/CflA family efflux MFS transporter	1 (5)	Bcr/CflA family efflux MFS transporter	1 (5)	Bcr/CflA family efflux MFS transporter	1 (5)	Bcr/CflA family efflux MFS transporter	1 (5)	Bcr/CflA family efflux MFS transporter	1 (5)	Bcr/CflA family efflux MFS transporter	1 (5)	Electrochemical Potential-driven transporters - Porters	1 (5)

**7**	WP_002 854281.1	uracil-DNA glycosylase	uracil-DNA glycosylase	1 (5)	uracil-DNA glycosylase	1 (5)	uracil-DNA glycosylase	1 (5)	uracil-DNA glycosylase	1 (5)	uracil-DNA glycosylase	1 (5)	uracil-DNA glycosylase	1 (5)	Transferases - Glycosyltransferases	1 (5)
**8**	WP_002 859393.1	acetylglutamate kinase	acetylglutamate kinase	1 (5)	acetylglutamate kinase	1 (5)	acetylglutamate kinase	1 (5)	acetylglutamate kinase	1 (5)	acetylglutamate kinase	1 (5)	acetylglutamate kinase	1 (5)	acetylglutamate kinase	1 (5)
**9**	WP_002 854336.1	molybdate ABC transporter permease	molybdate ABC transporter permease	1 (5)	molybdate ABC transporter permease	1 (5)	molybdate ABC transporter permease	1 (5)	molybdate ABC transporter permease	1 (5)	molybdate ABC transporter permease	1 (5)	molybdate ABC transporter permease	1 (5)	Electrochemical Potential-driven transporters	1 (4)
**10**	WP_002 859852.1	multidrug efflux SMR transporter	multidrug efflux SMR transporter	1 (5)	multidrug efflux SMR transporter	1 (5)	multidrug efflux SMR transporter	1 (5)	multidrug efflux SMR transporter	1 (5)	EamA-like transporter family	1 (4)	multidrug efflux SMR transporter	1 (5)	Metal-binding	1 (3)
**11**	WP_009 882032.1	prephenate dehydratase	prephenate dehydratase	1 (5)	prephenate dehydratase	1 (5)	prephenate dehydratase	1 (5)	prephenate dehydratase	1 (5)	prephenate dehydratase	1 (5)	Phosphate binding protein-like	1 (2)	Zinc-binding, Forming Carbon-Oxygen Bonds	1 (2)
**12**	WP_002 858694.1	lysine--tRNA ligase	tRNA synthetases	1 (5)	tRNA synthetases	1 (5)	tRNA synthetases	1 (5)	tRNA synthetases	1 (5)	tRNA synthetases	1 (5)	tRNA synthetases	1 (5)	Forming Carbon-Oxygen Bonds	1 (3)
**13**	WP_009 882169.1	YigZ family protein	UPF0029	1 (2)	UPF0029	1 (2)	UPF0029	1 (2)	Impact family	1 (2)	UPF0029	1 (2)	YigZ family protein	1 (5)	Transferring Phosphorus-Containing Groups	1 (2)
**14**	WP_002 857383.1	FAD-binding protein	FAD-binding protein	1 (5)	FAD-binding protein	1 (5)	FAD-binding protein	1 (5)	FAD-binding protein	1 (5)	Fumarate reductase flavoprotein C-term	1 (4)	FAD-binding protein	1 (5)	Acting on the CH-CH group of donors	1 (4)
**15**	WP_002 782934.1	ribosomal protein S12	ribosomal protein S12	1 (5)	ribosomal protein S12	1 (5)	ribosomal protein S12	1 (5)	ribosomal protein S12	1 (5)	ribosomal protein S12	1 (5)	Nucleic acid-binding proteins	0 (2)	rRNA-binding proteins	1 (4)
**16**	WP_002 857290.1	HIT domain-containing protein	HIT domain-containing protein	1 (5)	HIT domain-containing protein	1 (5)	HIT domain-containing protein	1 (5)	HIT domain-containing protein	1 (5)	HIT domain-containing protein	1 (5)	HIT domain-containing protein	1 (5)	Transferases - Acyltransferases,	1 (3)

														Phosphorus-Oxygen Lyases	
**17**	WP_002869243.1	ferrochelatase	ferrochelatase	1 (5)	ferrochelatase	1 (5)	ferrochelatase	1 (5)	ferrochelatase	1 (5)	ferrochelatase	1 (5)	ferrochelatase	1 (5)	Transferring Phosphorus-Containing Groups, All lipid-binding proteins	1 (3)
**18**	WP_002854879.1	flagellar basal body rod protein FlgB	No result	0 (2)	No result	0 (2)	No result	0 (2)	flagellar basal body rod protein FlgB	1 (5)	flagellar basal body rod protein FlgB	1 (5)	No result	0 (2)	Structural proteins	1 (4)
**19**	WP_002880964.1	endolytic transglycosylase MltG	endolytic transglycosylase MltG	1 (5)	endolytic transglycosylase MltG	1 (5)	endolytic transglycosylase MltG	1 (5)	endolytic transglycosylase MltG	1 (5)	endolytic transglycosylase MltG	1 (5)	No result	0 (2)	Transferases - Glycosyltransferases	1 (4)
**20**	WP_002856958.1	ATP-binding cassette domain-containing protein	ATP-binding cassette domain-containing protein	1 (5)	ATP-binding cassette domain-containing protein	1 (5)	ATP-binding cassette domain-containing protein	1 (5)	ATP-binding cassette domain-containing protein	1 (5)	ATP-binding cassette domain-containing protein	1 (5)	ATP-binding cassette domain-containing protein	1 (5)	Acting on Acid Anhydrides	1 (3)
**21**	WP_002868904.1	TolC family protein	TolC family protein	1 (5)	TolC family protein	1 (5)	TolC family protein	1 (5)	TolC family protein	1 (5)	TolC family protein	1 (5)	TolC family protein	1 (5)	Zinc-binding	0 (2)
**22**	WP_002869361.1	carbamoyltransferase HypF	carbamoyltransferase HypF	1 (5)	carbamoyltransferase HypF	1 (5)	carbamoyltransferase HypF	1 (5)	carbamoyltransferase HypF	1 (5)	carbamoyltransferase HypF	1 (5)	carbamoyltransferase HypF	1 (5)	Zinc-binding	0 (2)
**23**	WP_002869360.1	hydrogenase formation protein HypD	hydrogenase formation protein HypD	1 (5)	hydrogenase formation protein HypD	1 (5)	hydrogenase formation protein HypD	1 (5)	hydrogenase formation protein HypD	1 (5)	hydrogenase formation protein HypD	1 (5)	No result	0 (2)	Zinc-binding, Carbon-Carbon Lyases	1 (3)
**24**	WP_002869354.1	aspartate--tRNA ligase	aspartate--tRNA ligase	1 (5)	aspartate--tRNA ligase	1 (5)	aspartate--tRNA ligase	1 (5)	aspartate--tRNA ligase	1 (5)	aspartate--tRNA ligase	1 (5)	aspartate--tRNA ligase	1 (5)	Forming Carbon-Oxygen Bonds, Zinc-binding	1 (3)
**25**	WP_002869349.1	MFS transporter	Proton-dependent	1 (3)	MFS transporter	1 (5)	MFS transporter	1 (5)	MFS transporter	1 (5)	MFS transporter	1 (5)	MFS transporter	1 (5)	Iron-binding	1 (3)

			oligopeptide transporter													
**26**	WP_002869372.1	molecular chaperone DnaK	molecular chaperone DnaK	1 (5)	molecular chaperone DnaK	1 (5)	molecular chaperone DnaK	1 (5)	molecular chaperone DnaK	1 (5)	molecular chaperone DnaK	1 (5)	molecular chaperone DnaK	1 (5)	All lipid-binding proteins	1 (3)
**27**	WP_009882420.1	nucleotide exchange factor GrpE	nucleotide exchange factor GrpE	1 (5)	nucleotide exchange factor GrpE	1 (5)	nucleotide exchange factor GrpE	1 (5)	nucleotide exchange factor GrpE	1 (5)	nucleotide exchange factor GrpE	1 (5)	nucleotide exchange factor GrpE	1 (5)	Zinc-binding, Acting on Acid Anhydrides	1 (3)
**28**	WP_002857174.1	serine O-acetyltransferase	Hexapeptide repeat	1 (2)	Hexapeptide repeat	1 (2)	Hexapeptide repeat	1 (2)	serine O-acetyltransferase	1 (5)	Carbonic anhydrase or acetyltransferase, isoleucine patch superfamily	0 (2)	serine O-acetyltransferase	1 (5)	Phosphotransfer-driven group translocators , Manganese-binding	0 (2)
**29**	WP_011812734.1	ATP-dependent helicase	ATP-dependent helicase	1 (5)	ATP-dependent helicase	1 (5)	ATP-dependent helicase	1 (5)	ATP-dependent helicase	1 (5)	ATP-dependent helicase	1 (5)	ATP-dependent helicase	1 (5)	Zinc-binding, All DNA-binding	1 (3)
**30**	WP_002867950.1	alpha/beta hydrolase	alpha/beta hydrolase	1 (5)	alpha/beta hydrolase	1 (5)	alpha/beta hydrolase	1 (5)	alpha/beta hydrolase	1 (5)	alpha/beta hydrolase	1 (5)	alpha/beta hydrolase	1 (5)	All lipid-binding proteins	1 (3)
**31**	WP_002869103.1	c-type cytochrome	c-type cytochrome	1 (5)	c-type cytochrome	1 (5)	c-type cytochrome	1 (5)	c-type cytochrome	1 (5)	c-type cytochrome	1 (5)	c-type cytochrome	1 (5)	Zinc-binding	1 (3)
**32**	WP_011812744.1	DNA translocase FtsK	DNA translocase FtsK	1 (5)	DNA translocase FtsK	1 (5)	DNA translocase FtsK	1 (5)	DNA translocase FtsK	1 (5)	DNA translocase FtsK	1 (5)	DNA translocase FtsK	1 (5)	Zinc-binding, Acting on peptide bonds (Peptidases)	1 (3)
**33**	WP_002853404.1	GNAT family N-acetyltransferase	GNAT family N-acetyltransferase	1 (5)	GNAT family N-acetyltransferase	1 (5)	GNAT family N-acetyltransferase	1 (5)	GNAT family N-acetyltransferase	1 (5)	GNAT family N-acetyltransferase	1 (5)	GNAT family N-acetyltransferase	1 (5)	All lipid-binding proteins	1 (3)
**34**	WP_002853451.1	RNA polymerase sigma factor	RNA polymerase sigma factor	1 (5)	RNA polymerase sigma factor	1 (5)	RNA polymerase sigma factor	1 (5)	RNA polymerase sigma factor	1 (5)	RNA polymerase sigma factor RpoD	1 (5)	RNA polymerase sigma factor	1 (5)	DNA-directed RNA polymeras	1 (5)

		RpoD	RpoD		RpoD		RpoD		RpoD				RpoD		e	
**35**	WP_002 856550.1	potassium transporter TrkA	potassium transporter TrkA	1 (5)	potassium transporter TrkA	1 (5)	potassium transporter TrkA	1 (5)	potassium transporter TrkA	1 (5)	potassium transporter TrkA	1 (5)	potassium transporter TrkA	1 (5)	Glycosyltra nsferases, Acting on Ester Bonds	1 (3)
**36**	WP_002 852861.1	SsrA-binding protein SmpB	SsrA-binding protein SmpB	1 (5)	SsrA-binding protein SmpB	1 (5)	SsrA-binding protein SmpB	1 (5)	SsrA-binding protein SmpB	1 (5)	SsrA-binding protein SmpB	1 (5)	SsrA-binding protein SmpB	1 (5)	RNA-binding proteins	1 (3)
**37**	WP_002 855885.1	FAD-binding protein	FAD-binding protein	1 (5)	FAD-binding protein	1 (5)	FAD-binding protein	1 (5)	FAD-binding protein	1 (5)	FAD-binding protein	1 (5)	FAD-binding protein	1 (5)	Acting on the CH-OH group of donors, Manganes e-binding	1 (3)
**38**	WP_002 856003.1	riboflavin synthase	riboflavin synthase	1 (5)	riboflavin synthase	1 (5)	riboflavin synthase	1 (5)	riboflavin synthase	1 (5)	riboflavin synthase	1 (5)	riboflavin synthase	1 (5)	Transferrin g Alkyl or Aryl Groups, Other than Methyl Groups	1 (3)
**39**	WP_002 855731.1	bacteriohe merythrin	bacteriohe merythrin	1 (5)	bacteriohe merythrin	1 (5)	bacteriohe merythrin	1 (5)	bacteriohe merythrin	1 (5)	bacteriohemer ythrin	1 (5)	bacteriohe merythrin	1 (5)	Zinc-binding, All DNA-binding	1 (3)
**40**	WP_002 869409.1	GDP-L-fucose synthase	NAD dependent epimerase	0 (2)	GDP-L-fucose synthase	1 (5)	GDP-L-fucose synthase	1 (5)	GDP-L-fucose synthase	1 (5)	Short-chain dehydrogenas es/reductases (SDR)	0 (2)	AD(P)-binding Rossmann-fold domains	0 (2)	server error	0 (2)

S4 Table. Annotation dataset results for the 50 HPs submitted to the workflow with Pfam, SMART, MOTIF, INTERPROSCAN, CDART, SUPERFAMILY and SVMprot

No.	Protein ID	PFAM	SMART	MOTIF	INTERPROSCAN	CDART	SUPERFAMILY	SVMProt
1	WP_002868767.1	TolB amino-terminal domain	CsgG	Curli production assembly,transport component CsgG	Curli production assembly,transport component CsgG	TolB amino-terminal domain	CC0632-like	All lipid-binding proteins
2	WP_002854524.1	Chemotaxis phosphatase CheX	CheX	Chemotaxis phosphatase CheX	chemotaxiz phosphatase cheX- like domain	CheC-like family	CheC-like	Transferring Phosphorus-Containing Groups
3	WP_009882162.1	SprA-related family	SprA-related	SprA-related family	SprA-related family	SprA-related family	No result	Zinc-binding
4	WP_010790856.1	Pyridoxamine 5'-phosphate oxidase	Pyridox_oxidase	Pyridoxamine 5'-phosphate oxidase	Pyridoxamine 5'-phosphate oxidase	Pyridoxine 5'-phosphate (PNP) oxidase-like	PNP-oxidase like	Zinc-binding
5	WP_009882239.1	haemagglutination activity domain	Haemagg_act	haemagglutination activity domain	Filamentous haemagglutinin, N-terminal	haemagglutination activity domain	Filamentous hemagglutinin FhaB, secretion domain	All lipid-binding proteins
6	WP_002854991.1	FxsA cytoplasmic membrane protein, FxsA	FxsA	FxsA cytoplasmic membrane protein	FxsA cytoplasmic membrane protein	No result	No result	TC1.E Channels
7	WP_002855029.1	DNA replication regulator, HobA	HobA	DNA replication regulator	DNA replication regulator, HobA	DNA replication regulator, HobA Superfamily	SMI1	All lipid-binding proteins
8	WP_002868905.1	GDSL-like Lipase	Lipase_GDSL	GDSL-like Lipase	GDSL lipase	SGNH_hydrolase Superfamily/esterases and lipases	SGNH hydrolase/esterases and lipases	Zinc-binding
9	WP_002869356.1	Divergent polysaccharide deacetylase	Polysacc_deac_2	Divergent polysaccharide deacetylase	Divergent polysaccharide deacetylase	Divergent polysaccharide deacetylase	Divergent polysaccharide deacetylase	EC3.1 Hydrolases - Acting on Ester Bonds
10	WP_002856929.1	C4-type zinc ribbon domain	zf-RING_7	C4-type zinc ribbon domain	C4-type zinc ribbon domain	C4-type zinc ribbon domain	Tropomyosin	All DNA-binding
11	WP_002869028.1	Esterase-like activity of phytase	Phytase-like	Esterase-like activity of phytase	Phytase-like domain	SdiA-regulated Superfamily	No result	Zinc-binding
12	WP_011812736.1	DUF234	DUF234	DUF234	Domain of unknown function DUF234	DUF4143 Superfamily	Restriction endonuclease-like	Zinc-binding

**13**	WP_002868 809.1	Ankyrin repeats, Ank_2	Ankyrin repeats, Ank_2	Ankyrin repeats, Ank_2	Ankyrin repeat-containing domain	ANK Superfamily	Ankyrin repeat	EC3.2 Hydrolases - Glycosylases
**14**	WP_002869 368.1	Type-1V conjugative transfer system mating pair stabilisation, TraN	TraN	Type-1V conjugative transfer system mating pair stabilisation, TraN	Type-F conjugative transfer system mating-pair stabilisation protein TraN	Type-1V conjugative transfer system mating pair stabilisation, TraN Superfamily	TB module	All lipid-binding proteins
**15**	WP_009882 583.1	NLPC_P60	NLPC_P60	NLPC_P60 stabilising domain, N term	NLPC/P60, N-terminal domain	NlpC/P60 family	NlpC/P60	Forming Carbon-Oxygen Bonds
**16**	WP_002853 389.1	Jag N-terminus	Jag_N	Jag N-terminus	Jag, N-terminal domain superfamily	Jag N-terminus	No result	Transferring One-Carbon Groups
**17**	WP_009882 608.1	Adhesin from Campylobacter	JLPA	Adhesin from Campylobacter	Adhesin JlpA, Campylobacter	JLPA Superfamily , Adhesin from Campylobacter	No result	Zinc-binding
**18**	WP_002856 369.1	Putative beta-lactamase-inhibitor-like	PepSY_like	Putative beta-lactamase-inhibitor-like	Putative beta-lactamase-inhibitor-like, PepSY-like	Putative beta-lactamase-inhibitor-like, PepSY-like	BT0923-like	Sodium-binding
**19**	WP_079254 190.1	Beta-1,4-N-acetylgalactosaminyltransferase (CgtA)	CgtA	Beta-1,4-N-acetylgalactosaminyltransferase (CgtA)	Beta-1,4-N-acetylgalactosaminyltransferase	Beta-1,4-N-acetylgalactosaminyltransferase (CgtA)	No result	Magnesium-binding
**20**	WP_002856 180.1	No result	HMA	Heavy-metal-associated domain	Heavy metal-associated domain	Heavy-metal-associated domain (HMA)	HMA, heavy metal-associated domain	Copper-binding
**21**	WP_002831 611.1	Transcription factor zinc-finger	zf-TFIIB	Transcription factor zinc-finger	Transcription factor zinc-finger	Transcription factor zinc-finger	No result	Magnesium-binding
**22**	WP_002790 076.1	Methyl-accepting chemotaxis protein (MCP) signalling domain	Methyl-accepting chemotaxis-like domains	Methyl-accepting chemotaxis protein (MCP) signalling domain	Methyl-accepting chemotaxis protein (MCP) signalling domain	Methyl-accepting chemotaxis protein (MCP) signalling domain	Methyl-accepting chemotaxis protein (MCP) signaling domain	P-P-bond-hydrolysis-driven transporters
**23**	WP_002853 792.1	Plasminogen-binding protein pgbA N-terminal	PGBA_N	Plasminogen-binding protein pgbA N-terminal	Plasminogen-binding protein PgbA, N-terminal	Plasminogen-binding protein pgbA N-terminal	No result	Manganese-binding
**24**	WP_002869 072.1	Putative S-adenosyl-L-methionine-dependent methyltransferase	Methyltransf_28	Putative S-adenosyl-L-methionine-dependent methyltransferase	S-adenosyl-L-methionine-dependent methyltransferase	SAM-dependent methyltransferase, MidA family	S-adenosyl-L-methionine-dependent methyltransferases	Zinc-binding
**25**	WP_002869 097.1	MaoC like domain	MaoC_dehydratas	MaoC like domain	MaoC-like dehydratase domain	Short-chain dehydrogenases	MaoC-like	Transferring Phosphorus-

								Containing Groups
**26**	WP_002869 326.1	Carboxypeptidase controlling helical cell shape catalytic	Peptidase_M 99	Carboxypeptidase controlling helical cell shape catalytic	Metallo-carboxypeptidase, C-terminal domain	C-terminal domain of metallo-carboxypeptidase	Zn-dependent exopeptidases	Zinc-binding
**27**	WP_002869 139.1	Pyruvate phosphate dikinase, PEP	PPDK_N	Pyruvate phosphate dikinase, PEP	Pyruvate phosphate dikinase, PEP	Pyruvate phosphate dikinase, PEP	Pyruvate phosphate dikinase, N-terminal domain	Forming Carbon-Oxygen Bonds
**28**	WP_002869 194.1	No result	Rod-binding	Rod binding protein	Uncharacterised conserved protein UCP007248	Mannosyl-glycoprotein endo-beta-N-acetylglucosaminidase	No result	Acting on peptide bonds (Peptidases)
**29**	WP_002869 195.1	Anti-sigma-28 factor	FlgM	Anti-sigma-28 factor	Anti-sigma-28 factor FlgM superfamily	No result	Anti-sigma factor FlgM	Sodium-binding
**30**	WP_002856 630.1	PD-(D/E)XK nuclease superfamily	PDDEXK_1	PD-(D/E)XK nuclease superfamily, PDDEXK_1	PD-(D/E)XK endonuclease-like domain, AddAB-type	Inactivated superfamily I helicase	P-loop containing nucleoside triphosphate hydrolases	Forming Carbon-Oxygen Bonds
**31**	WP_002855 458.1	MgtE intracellular N domain	MgtE_N	MgtE intracellular N domain	Magnesium transporter, MgtE intracellular domain	Flagellar motility protein MotE, a chaperone for MotC folding	MgtE N-terminal domain-like	ATP-binding cassette (ABC) family
**32**	WP_002797 496.1	Flagellar FliJ protein	FliJ	Flagellar FliJ protein	Flagellar export FliJ	Flagellar FliJ protein	No result	Magnesium-binding
**33**	WP_024088 174.1	Nitrate reductase delta subunit	Nitrate_red_ del	Nitrate reductase delta subunit	Nitrate reductase chaperone	Nitrate reductase delta subunit	TorD-like	Type II (general) secretory pathway (IISP) family
**34**	WP_009883 030.1	AAA domain, putative AbiEii toxin, Type IV TA system, AAA_21	AAA_21	AAA domain, putative AbiEii toxin, Type IV TA system, AAA_21	ATPase, AAA-type, core	AAA domain, putative AbiEii toxin, Type IV TA system, AAA_21 Superfamily	ABC transporter ATPase domain-like	Glycosyltransf erases
**35**	WP_002824 979.1	putative NADH-ubiquinone oxidoreductase chain E	NADH-UOR_E	putative NADH-ubiquinone oxidoreductase chain E	Putative NADH-ubiquinone oxidoreductase chain E	putative NADH-ubiquinone oxidoreductase chain E	SirA-like	All lipid-binding proteins
**36**	WP_002869 225.1	DMSO reductase anchor subunit (DmsC)	DmsC	DMSO reductase anchor subunit (DmsC)	DMSO reductase anchor subunit (DmsC)	DMSOR_beta-like Superfamily	No result	Acting on a heme group of donors
**37**	WP_002856 602.1	Putative beta-lactamase-inhibitor-like	PepSY_like	Putative beta-lactamase-inhibitor-like	Putative beta-lactamase-inhibitor-like, PepSY-like	Putative beta-lactamase-inhibitor-like, PepSY-like	BT0923-like	All lipid-binding proteins

**38**	WP_002868888.1	No result	TPR_2/ TPR_8	Tetratricopeptide repeat, TPR_2	Tetratricopeptide repeat	Lipopolysaccharide biosynthesis regulator YciM, contains six TPR domains and a predicted metal-binding C-terminal domain	Tetratricopeptide repeat (TPR)	Zinc-binding
**39**	WP_002868880.1	ABC-type transport auxiliary lipoprotein component	ABC_trans_aux	ABC-type transport auxiliary lipoprotein component	ABC-type transport auxiliary lipoprotein component	ABC-type transport auxiliary lipoprotein component	XCC0632-like	Carbon-Oxygen Lyases
**40**	WP_009883121.1	Flagellar FLiS export co-chaperone	FliS_cochap	Flagellar FLiS export co-chaperone, HP1076	Flagellar FLiS export co-chaperone, HP1076	Flagellar FLiS export co-chaperone, HP1076	No result	All DNA-binding
**41**	WP_002860117.1	Menaquinone biosynthesis	VitK2_biosynth	Menaquinone biosynthesis	Menaquinone biosynthesis enzyme	member of the type 2 periplasmic binding fold protein superfamily	Periplasmic binding protein-like II	Glycosyltransferases
**42**	WP_002779704.1	T-antigen specific domain	Papo_T_antigen	T-antigen specific domain	Small/middle T-antigen superfamily	No result	T-antigen specific domain-like	Acting on iron-sulfur proteins as donors
**43**	WP_011187233.1	Toprim domain	TOPRIM	Toprim domain	twinkle, TOPRIM domain	Uncharacterized domain associated with phage	No result	Zinc-binding
**44**	WP_011187235.1	AAA domain, AAA_25	AAA_25	AAA domain, AAA_25	P-loop containing nucleoside triphosphate hydrolase	TOPRIM Superfamily	P-loop containing nucleoside triphosphate hydrolases	Acting on Ester Bonds
**45**	WP_002809111.1	TrbM	TrbM	TrbM	TrbM	TrbM Superfamily	No result	Acting on a sulfur group of donors
**46**	WP_011117548.1	VirB8 protein	VirB8	VirB8 protein	Bacterial virulence protein VirB8	Virulence protein VirB8	VirB8-like	Metal-binding
**47**	WP_011117549.1	Conjugal transfer protein	CagX	Conjugal transfer protein	CagX	CagX	No result	Glycosyltransferases
**48**	WP_011117575.1	Type IV secretion system proteins,T4SS	T4SS	Type IV secretion system proteins, T4SS	Type IV secretion system, VirB5	VirB5 protein family	Type IV secretion system protein TraC	Type IV secretion system protein TraC
**49**	WP_011799393.1	TrbM	TrbM	TrbM	TrbM	TrbM	No result	Acting on Ester Bonds
**50**	WP_011117588.1	PemK-like, MazF-like toxin of type II toxin-antitoxin system	PemK_toxin	PemK-like, MazF-like toxin of type II toxin-antitoxin system	mRNA interferase PemK-like	PemK-like, MazF-like toxin of type II toxin-antitoxin system	PemK	All DNA-binding

S5 Table. Results of the blastp search for similar sequences against non-reduntant (nr) database

No.	Protein ID	*Organism*	Query cover	e-value	Score (bits)	Identity	Product
1	WP_002868767.1	*Campylobacter jejuni subsp. jejuni 129-258*	100%	0.0	798	100%	hypothetical protein
		Campylobacter jejuni CVM 41974	100%	0.0	795	99%	hypothetical protein
		Campylobacter jejuni BJ-CJGB95377	100%	0.0	793	99%	hypothetical protein
		Campylobacter jejuni subsp. jejuni 84-25	100%	0.0	793	99%	hypothetical protein
		Campylobacter jejuni X	100%	0.0	792	99%	hypothetical protein
2	WP_002854524.1	*Campylobacter jejuni subsp. jejuni 327*	100%	5.00E-97	285	100%	hypothetical protein
		Campylobacter jejuni subsp. jejuni CG8486	100%	6.00E-97	284	100%	hypothetical protein
		Campylobacter jejuni subsp. jejuni M1	100%	7.00E-97	284	100%	hypothetical protein
		Campylobacter jejuni RM1221	100%	1.00E-96	283	100%	hypothetical protein
		Campylobacter jejuni subsp. jejuni LMG 23211	100%	3.00E-96	282	99%	hypothetical protein
3	WP_009882162.1	*Campylobacter jejuni subsp. jejuni 81-176-DRH212*	100%	6.00E-174	487	100%	hypothetical protein
		Campylobacter sp. BCW_4319	100%	1.00E-172	484	99%	hypothetical protein
		Campylobacter sp. BCW_4319	100%	3.00E-171	480	97%	hypothetical protein
		Campylobacter jejuni subsp. jejuni CF93-6	100%	3.00E-169	475	97%	hypothetical protein
4	WP_010790856.1	*Campylobacter jejuni subsp. jejuni*	100%	1.00E-96	283	99%	pyridoxamine 5'-phosphate oxidase
		Campylobacter sp. 109	100%	3.00E-96	282	99%	pyridoxamine 5'-phosphate oxidase
		Campylobacter sp. 3	100%	5.00E-96	281	99%	pyridoxamine 5'-phosphate oxidase
		Campylobacter sp. BCW_8713	100%	6.00E-96	281	99%	pyridoxamine 5'-phosphate oxidase
		Campylobacter jejuni subsp. doylei	100%	6.00E-96	281	99%	pyridoxamine 5'-phosphate oxidase
5	WP_009882239.1	*Campylobacter jejuni subsp. jejuni*	100%	0.0	1096	99%	filamentous hemagglutinin N-terminal domain-containing protein
		Campylobacter jejuni BJ-CJGB96114	100%	0.0	1093	99%	filamentous hemagglutinin N-terminal domain-containing protein
		Campylobacter jejuni subsp. jejuni 81-176-UMCW9	100%	0.0	1093	99%	filamentous hemagglutinin N-terminal domain-containing protein

**6**	WP_002854991.1	*Campylobacter jejuni RM1221*	100%	8.00E-83	247	99%	integral membrane protein
		Campylobacter jejuni subsp. jejuni 260.94	100%	1.00E-82	247	99%	integral membrane protein
		Campylobacter jejuni subsp. jejuni HB93-13	100%	2.00E-82	246	99%	integral membrane protein
		Campylobacter jejuni subsp. jejuni D2600	100%	2.00E-82	246	99%	integral membrane protein
		Campylobacter jejuni subsp. jejuni LMG 23264	100%	2.00E-82	246	98%	integral membrane protein
**7**	WP_002855029.1	*Campylobacter jejuni subsp. jejuni 260.94*	100%	2.00E-124	357	99%	hypothetical protein
		Campylobacter jejuni subsp. jejuni ICDCCJ07001	100%	6.00E-124	355	99%	hypothetical protein
		Campylobacter jejuni subsp. jejuni 129-258	100%	6.00E-124	355	99%	hypothetical protein
		Campylobacter jejuni K1	100%	6.00E-124	355	98%	hypothetical protein
**8**	WP_002868905.1	*Campylobacter jejuni subsp. jejuni 129-258*	100%	0.0	787	100%	hypothetical protein
		Campylobacter jejuni subsp. jejuni 81-176-DRH212	100%	0.0	786	99%	hypothetical protein
		Campylobacter jejuni subsp. jejuni CG8486	100%	0.0	784	99%	hypothetical protein
**9**	WP_002869356.1	*Campylobacter jejuni subsp. jejuni 129-258*	100%	0.0	718	99%	polysaccharide deacetylase
		Campylobacter jejuni subsp. jejuni 2008-872	100%	0.0	716	99%	polysaccharide deacetylase
		Campylobacter jejuni subsp. jejuni 1997-11	100%	0.0	716	99%	polysaccharide deacetylase
		Campylobacter jejuni subsp. jejuni 1798	100%	0.0	716	99%	polysaccharide deacetylase
		Campylobacter jejuni K1	100%	0.0	716	99%	polysaccharide deacetylase
**10**	WP_002856929.1	*Campylobacter jejuni RM1221*	100%	3.00E-167	470	100%	zinc ribbon domain protein
		Campylobacter jejuni subsp. jejuni S3	100%	1.00E-166	468	99%	zinc ribbon domain protein
		Campylobacter jejuni subsp. jejuni 327	100%	1.00E-166	468	99%	zinc ribbon domain protein
		Campylobacter jejuni CVM 41910	100%	1.00E-166	468	99%	zinc ribbon domain protein
		Campylobacter jejuni CVM 41927	100%	2.00E-166	468	99%	zinc ribbon domain protein
**11**	WP_002869028.1	*Campylobacter jejuni subsp. jejuni 81-*	100%	0.0	884	100%	glycerophosphodiester

		176-DRH212					phosphodiesterase
		Campylobacter jejuni subsp. jejuni 81-176-UMCW7	100%	0.0	883	99%	glycerophosphodiester phosphodiesterase
		Campylobacter sp. BCW_4319	100%	0.0	882	99%	glycerophosphodiester phosphodiesterase
		Campylobacter sp. 1	100%	0.0	882	99%	glycerophosphodiester phosphodiesterase
		Campylobacter jejuni 30318	100%	0.0	881	99%	glycerophosphodiester phosphodiesterase
**12**	WP_011812736.1	*Campylobacter sp. 114*	100%	0.0	565	100%	hypothetical protein
		Campylobacter sp. BCW_8709	100%	0.0	565	99%	hypothetical protein
		Campylobacter jejuni subsp. jejuni 260.94	100%	0.0	564	99%	hypothetical protein
		Campylobacter jejuni subsp. jejuni ICDCCJ07001	100%	0.0	563	99%	hypothetical protein
**13**	WP_002868809.1	*Campylobacter jejuni subsp. jejuni 129-258*	100%	0.0	816	100%	ankyrin repeat-containing protein
		Campylobacter jejuni subsp. jejuni 81-176-UMCW7	100%	0.0	815	99%	ankyrin repeat-containing protein
		Campylobacter jejuni subsp. jejuni 1213	100%	0.0	814	99%	ankyrin repeat-containing protein
		Campylobacter jejuni K1	100%	0.0	813	99%	ankyrin repeat-containing protein
		Campylobacter jejuni subsp. jejuni 1997-11	100%	0.0	813	99%	ankyrin repeat-containing protein
**14**	WP_002869368.1	*Campylobacter jejuni subsp. jejuni 81-176-DRH212*	100%	7.00E-103	300	100%	mating pair stabilization protein
		Campylobacter sp. BCW_4319	100%	2.00E-101	296	98%	mating pair stabilization protein
		Campylobacter jejuni subsp. jejuni HB93-13	100%	1.00E-100	294	98%	mating pair stabilization protein
		Campylobacter jejuni K1	100%	2.00E-100	294	98%	mating pair stabilization protein
		Campylobacter jejuni CVM 41974	100%	4.00E-100	293	97%	mating pair stabilization protein
**15**	WP_009882583.1	*Campylobacter jejuni subsp. jejuni LMG 9217*	100%	0.0	905	99%	SH3_6 and SH3_7 domain-containing protein
		Campylobacter sp. BCW_7460	100%	0.0	904	99%	SH3_6 and SH3_7 domain-containing protein
		Campylobacter jejuni subsp. jejuni 129-258	100%	0.0	904	99%	SH3_6 and SH3_7 domain-containing protein

		Campylobacter sp. 1	100%	0.0	902	99%	SH3_6 and SH3_7 domain-containing protein
		Campylobacter jejuni subsp. jejuni 51494	100%	0.0	902	99%	SH3_6 and SH3_7 domain-containing protein
**16**	WP_002853389.1	*Campylobacter jejuni subsp. jejuni CF93-6*	100%	0.0	551	99%	RNA-binding protein
		Campylobacter jejuni subsp. jejuni 84-25	100%	0.0	550	99%	RNA-binding protein
		Campylobacter jejuni subsp. jejuni 1336	100%	0.0	550	99%	RNA-binding protein
		Campylobacter jejuni subsp. jejuni IA3902	100%	0.0	550	99%	RNA-binding protein
		Campylobacter jejuni subsp. jejuni 305	100%	0.0	550	99%	RNA-binding protein
**17**	WP_009882608.1	*Campylobacter jejuni subsp. jejuni 81-176-UMCW7*	100%	0.0	727	99%	lipoprotein
		Campylobacter jejuni subsp. jejuni 81-176-UMCW9	100%	0.0	727	99%	lipoprotein
		Campylobacter sp. BCW_4319	100%	0.0	725	99%	lipoprotein
		Campylobacter jejuni subsp. jejuni HB93-13	100%	0.0	724	99%	lipoprotein
		Campylobacter jejuni subsp. jejuni 2008-894	100%	0.0	724	99%	lipoprotein
**18**	WP_002856369.1	*Campylobacter jejuni subsp. jejuni 260.94*	100%	3.00E-92	272	99%	Putative beta-lactamase-inhibitor-like, PepSY-like
		Campylobacter jejuni subsp. jejuni 81116	100%	1.00E-91	271	99%	Putative beta-lactamase-inhibitor-like, PepSY-like
		Campylobacter jejuni subsp. jejuni M1	100%	2.00E-91	270	99%	Putative beta-lactamase-inhibitor-like, PepSY-like
		Campylobacter jejuni subsp. jejuni ICDCCJ07001	100%	3.00E-91	270	99%	Putative beta-lactamase-inhibitor-like, PepSY-like
		Campylobacter jejuni subsp. jejuni 327	100%	6.00E-91	269	99%	Putative beta-lactamase-inhibitor-like, PepSY-like
**19**	WP_079254190.1	*Campylobacter jejuni subsp. jejuni 81-176*	100%	8.00E-31	110	100%	hypothetical protein
		Campylobacter sp. US54	100.00%	8.00E-31	110	100%	hypothetical protein
**20**	WP_002856180.1	*Campylobacter jejuni subsp. jejuni 81-176-DRH212*	100%	6.00E-36	124	100%	heavy-metal-associated domain
		Campylobacter jejuni subsp. jejuni 81-176-UMCW7	100%	2.00E-35	123	98.44%	heavy-metal-associated domain

		Campylobacter jejuni CVM 41974	100%	3.00E-35	122	96.88%	heavy-metal-associated domain
		Campylobacter sp. BCW_4319	100%	8.00E-35	121	96.88%	heavy-metal-associated domain
		Campylobacter sp. CH278	100%	9.00E-35	121	96.88%	heavy-metal-associated domain
**21**	WP_002831611.1	*Campylobacter jejuni subsp. jejuni LMG 23211*	100%	2.00E-59	185	100%	hypothetical protein
		Campylobacter jejuni subsp. jejuni LMG 9872	100%	3.00E-59	185	98.85%	hypothetical protein
		Campylobacter sp. CH186	100%	3.00E-59	185	98.85%	hypothetical protein
		Campylobacter sp. US55	100%	6.00E-59	184	98.85%	hypothetical protein
		Campylobacter sp. US53	100%	7.00E-59	184	98.85%	hypothetical protein
**22**	WP_002790076.1	*Campylobacter jejuni subsp. jejuni 81-176*	100%	0.0	919	100.00%	methyl-accepting chemotaxis protein
		Campylobacter jejuni subsp. jejuni LMG 9879	100%	0.0	918	99.56%	methyl-accepting chemotaxis protein
		Campylobacter jejuni subsp. jejuni 2008-1025	100%	0.0	918	99.78%	methyl-accepting chemotaxis protein
		Campylobacter jejuni subsp. jejuni 260.94	100%	0.0	918	99.78%	methyl-accepting chemotaxis protein
**23**	WP_002853792.1	*Campylobacter jejuni CVM 41973*	100%	1.00E-173	486	100.00%	exporting protein
		Campylobacter jejuni CVM 41910	100%	2.00E-173	486	99.59%	exporting protein
		Campylobacter jejuni CVM 41922	100%	2.00E-173	486	99.59%	exporting protein
		Campylobacter jejuni CVM 41914	100%	3.00E-173	485	99.59%	exporting protein
		Campylobacter jejuni CVM 41936	100%	6.00E-173	484	99.59%	exporting protein
**24**	WP_002869072.1	*Campylobacter sp. BCW_4319*	100%	0.0	629	100.00%	hypothetical protein
		Campylobacter jejuni subsp. jejuni str. RM3420	100%	0.0	627	99.68%	hypothetical protein
		Campylobacter jejuni BJ-CJD101	100%	0.0	627	99.68%	hypothetical protein
		Campylobacter jejuni CVM 41974	100%	0.0	623	99.37%	hypothetical protein
		Campylobacter jejuni subsp. jejuni 140-16	100%	0.0	621	98.10%	hypothetical protein
**25**	WP_002869097.1	*Campylobacter jejuni subsp. jejuni 81-176*	100%	0.0	895	100.00%	MaoC like domain
		Campylobacter sp. BCW_4319	100%	0.0	892	99.78%	MaoC like domain
		Campylobacter sp. US54	100%	0.0	892	99.78%	MaoC like domain

		Campylobacter coli LMG 23342	100%	0.0	891	99.34%	MaoC like domain
		Campylobacter coli 15-537360	100%	0.0	890	99.56%	MaoC like domain
**26**	WP_002869326.1	*Campylobacter jejuni CVM 41974*	100%	0.0	945	100.00%	deacylase
		Campylobacter jejuni subsp. jejuni	100%	0.0	940	99.35%	deacylase
		Campylobacter sp. US12a	100%	0.0	940	99.35%	deacylase
		Campylobacter sp. CH165	100%	0.0	939	99.35%	deacylase
		Campylobacter sp. CH278	100%	0.0	939	99.35%	deacylase
**27**	WP_002869139.1	*Campylobacter jejuni subsp. jejuni 129-258*	100%	0.0	1579	100.00%	hypothetical protein
		Campylobacter sp. US54	100%	0.0	1576	99.74%	hypothetical protein
		Campylobacter jejuni 30318	100%	0.0	1573	99.61%	hypothetical protein
		Campylobacter jejuni subsp. jejuni 53161	100%	0.0	1572	99.49%	hypothetical protein
		Campylobacter jejuni subsp. jejuni 87459	100%	0.0	1571	99.49%	hypothetical protein
**28**	WP_002869195.1	*Campylobacter jejuni subsp. jejuni 129-258*	100%	5.00E-37	127	100.00%	flagellar biosynthesis anti-sigma factor FlgM
		Campylobacter sp. US54	100%	1.00E-36	126	98.46%	flagellar biosynthesis anti-sigma factor FlgM
		Campylobacter jejuni subsp. jejuni M129	100%	2.00E-36	125	98.46%	flagellar biosynthesis anti-sigma factor FlgM
		Campylobacter jejuni subsp. jejuni 1336	100%	3.00E-36	125	96.92%	flagellar biosynthesis anti-sigma factor FlgM
		Campylobacter coli 2680	100%	5.00E-36	124	96.92%	flagellar biosynthesis anti-sigma factor FlgM
**29**	WP_002856630.1	*Campylobacter jejuni subsp. jejuni str. RM3420*	100%	0.0	1560	100.00%	helicase AddB
		Campylobacter jejuni K5	100%	0.0	1559	99.87%	helicase AddB
		Campylobacter jejuni subsp. jejuni 1577	100%	0.0	1557	99.87%	helicase AddB
**30**	WP_002855458.1	*Campylobacter jejuni RM1221*	100%	6.00E-117	337	100.00%	nucleosidase
		Campylobacter jejuni subsp. jejuni S3	100%	9.00E-117	337	99.42%	nucleosidase
		Campylobacter jejuni Cj2	100%	2.00E-116	336	99.42%	nucleosidase
		Campylobacter jejuni 20176	100%	2.00E-116	336	99.42%	nucleosidase

		Campylobacter jejuni CVM 41922	100%	2.00E-116	336	99.42%	nucleosidase
**31**	WP_002797496.1	*Campylobacter jejuni RM1221*	100%	2.00E-95	280	100.00%	hypothetical protein
		Campylobacter coli 15-537360	100%	6.00E-95	279	99.30%	hypothetical protein
		Campylobacter jejuni subsp. jejuni R14	100%	8.00E-95	279	99.30%	hypothetical protein
		Campylobacter jejuni subsp. jejuni M129]	100%	8.00E-95	279	99.30%	hypothetical protein
**32**	WP_024088174.1	*Campylobacter jejuni subsp. jejuni M129*	100%	6.00E-167	469	100.00%	formate dehydrogenase-specific chaperone
		Campylobacter jejuni subsp. jejuni IA3902	100%	2.00E-166	468	99.58%	formate dehydrogenase-specific chaperone
		Campylobacter jejuni subsp. jejuni S3	100%	3.00E-166	467	99.58%	formate dehydrogenase-specific chaperone
		Campylobacter jejuni subsp. jejuni D42a	100%	5.00E-166	467	99.58%	formate dehydrogenase-specific chaperone
		Campylobacter sp. 112	100%	5.00E-166	467	99.58%	formate dehydrogenase-specific chaperone
**33**	WP_009883030.1	*Campylobacter jejuni subsp. jejuni 81-176-UMCW7*	100%	0.0	865	100.00%	ATP/GTP-binding protein
		Campylobacter jejuni subsp. jejuni 81-176	100%	0.0	865	100.00%	ATP/GTP-binding protein
		Campylobacter coli CVM 41944	100%	0.0	865	100.00%	ATP/GTP-binding protein
		Peptoniphilus sp. HMSC075B08	100%	0.0	863	99.77%	ATP/GTP-binding protein
		Streptococcus phocae	100%	0.0	862	99.77%	ATP/GTP-binding protein
**34**	WP_002824979.1	*Campylobacter jejuni subsp. doylei*	100%	5.00E-45	148	100.00%	NADH-ubiquinone oxidoreductase
		Campylobacter jejuni subsp. jejuni 81116	100%	9.00E-45	147	98.67%	NADH-ubiquinone oxidoreductase
		Campylobacter jejuni subsp. jejuni S3	100%	9.00E-45	147	98.67%	NADH-ubiquinone oxidoreductase
		Campylobacter jejuni subsp. jejuni PT14	100%	9.00E-45	147	98.67%	NADH-ubiquinone oxidoreductase
		Campylobacter coli RM4661	100%	9.00E-45	147	98.67%	NADH-ubiquinone oxidoreductase
**35**	WP_002869225.1	*Campylobacter sp. BCW_4319*	100%	0.0	577	100.00%	dimethylsulfoxide reductase
		Campylobacter jejuni subsp. jejuni 81-176	100%	0.0	575	99.65%	dimethylsulfoxide reductase
		Campylobacter jejuni subsp. jejuni str. RM3420	100%	0.0	574	99.65%	dimethylsulfoxide reductase

		Campylobacter jejuni subsp. jejuni HB93-13	100%	0.0	574	99.65%	dimethylsulfoxide reductase
		Campylobacter jejuni CVM 41974	100%	0.0	573	99.31%	dimethylsulfoxide reductase
**36**	WP_002856602.1	*Campylobacter jejuni subsp. jejuni 81116*	100%	2.00E-94	278	100%	periplasmic protein
		Campylobacter jejuni subsp. jejuni M1	100%	3.00E-94	277	99.28%	periplasmic protein
		Campylobacter jejuni subsp. jejuni ICDCCJ07001	100%	3.00E-94	277	99.28%	periplasmic protein
		Campylobacter jejuni subsp. jejuni str. RM3420	100%	5.00E-94	276	99.28%	periplasmic protein
		Campylobacter jejuni subsp. jejuni 1336	100%	5.00E-94	276	98.55%	periplasmic protein
**37**	WP_002868888.1	*Campylobacter jejuni subsp. jejuni S3*	100%	0.0	655	100.00%	periplasmic protein
		Campylobacter sp. BCW_6461	100%	0.0	654	99.70%	periplasmic protein
		Campylobacter sp. BCW_4319	100%	0.0	654	99.70%	periplasmic protein
		Campylobacter sp. BCW_6871	100%	0.0	654	99.70%	periplasmic protein
		Campylobacter jejuni subsp. jejuni 1213	100%	0.0	653	99.70%	periplasmic protein
**38**	WP_002868880.1	*Campylobacter jejuni subsp. jejuni 260.94*	100%	3.00E-139	395	100%	ABC transporter
		Campylobacter jejuni subsp. jejuni 81-176	100%	1.00E-138	394	98.99%	ABC transporter
		Campylobacter jejuni K1	100%	2.00E-138	394	99.50%	ABC transporter
		Campylobacter sp. BCW_4319	100%	2.00E-138	394	99.50%	ABC transporter
		Campylobacter sp. US54	100%	2.00E-138	394	99.50%	ABC transporter
**39**	WP_009883121.1	*Campylobacter jejuni subsp. jejuni 81-176*	100%	4.00E-116	335	100.00%	hypothetical protein
		Campylobacter jejuni 32488	100%	9.00E-116	334	99.40%	hypothetical protein
		Campylobacter jejuni subsp. jejuni 260.94	100%	2.00E-115	333	98.80%	hypothetical protein
		Campylobacter jejuni subsp. jejuni 1854	100%	2.00E-115	333	98.80%	hypothetical protein
		Campylobacter jejuni subsp. jejuni 1893	100%	3.00E-115	332	98.80%	hypothetical protein
**40**	WP_002860117.1	*Campylobacter jejuni CVM 41927*	100%	0.0	583	100.00%	S-ribosylhomocysteine lyase

		Campylobacter jejuni CVM 41900	100%	0.0	582	99.65%	S-ribosylhomocysteine lyase
		Campylobacter jejuni subsp. jejuni 1997-1	100%	0.0	582	99.65%	S-ribosylhomocysteine lyase
		Campylobacter sp. CH246	100%	0.0	582	99.65%	S-ribosylhomocysteine lyase
		Campylobacter sp. BCW_6461	100%	0.0	582	99.65%	S-ribosylhomocysteine lyase
**41**	WP_002779704.1	*Campylobacter jejuni subsp. jejuni 81-176*	100%	5.00E-56	176	100.00%	cpp11 like protein
		Campylobacter jejuni subsp. jejuni S3	100%	8.00E-56	176	100.00%	cpp11 like protein
		Campylobacter coli CVM N29710	100%	3.00E-55	175	98.86%	cpp11 like protein
		Campylobacter coli 2553	98%	6.00E-55	174	100.00%	cpp11 like protein
		Campylobacter coli 86119	100%	1.00E-54	173	97.73%	cpp11 like protein
**42**	WP_011187233.1	*Campylobacter jejuni subsp. jejuni 81-176*	100%	0.0	828	100%	cpp22 like protein
		Campylobacter jejuni subsp. jejuni 81-176	100%	0.0	827	99.75%	cpp22 like protein
		Campylobacter coli 1148	100%	0.0	827	99.75%	cpp22 like protein
		Campylobacter jejuni CVM 41946	100%	0.0	826	99.75%	cpp22 like protein
		Campylobacter sp. BCW_6462	100%	0.0	826	99.51%	cpp22 like protein
**43**	WP_011187235.1	*Campylobacter jejuni subsp. jejuni 81-176*	100%	0.0	1196	100.00%	cpp26 like protein
		Campylobacter jejuni subsp. jejuni M129	100%	0.0	1195	99.83%	cpp26 like protein
		Campylobacter jejuni subsp. jejuni D42a	100%	0.0	1193	99.83%	cpp26 like protein
**44**	WP_002809111.1	Campylobacter jejuni subsp. jejuni 81-176	100%	0.0	524	100.00%	conjugal transfer protein TrbM
		Campylobacter coli 1148	100%	0.0	523	99.61%	conjugal transfer protein TrbM
		Campylobacter coli 317/04	100%	0.0	523	99.61%	conjugal transfer protein TrbM
		Campylobacter sp. BCW_6462	100%	0.0	522	99.21%	conjugal transfer protein TrbM
		Campylobacter jejuni CVM 41934	100%	0.0	521	99.61%	conjugal transfer protein TrbM
**45**	WP_011117548.1	Campylobacter jejuni subsp. jejuni IA3902	100%	3.00E-162	456	100.00%	virulence protein
		Campylobacter jejuni subsp. jejuni 81-176	100%	9.00E-162	455	99.56%	virulence protein

		Campylobacter coli 132-6	100%	2.00E-161	454	99.56%	virulence protein
		Campylobacter jejuni X	100%	4.00E-160	451	98.67%	virulence protein
		Campylobacter coli RM1875	100%	6.00E-159	448	97.78%	virulence protein
**46**	WP_011117549.1	Campylobacter jejuni subsp. jejuni 81-176	100%	0.0	727	100.00%	type IV secretion system protein VirB9
		Campylobacter jejuni subsp. jejuni IA3902	100%	0.0	725	99.72%	type IV secretion system protein VirB10
		Campylobacter coli	100%	0.0	725	99.72%	type IV secretion system protein VirB11
**47**	WP_011117575.1	Campylobacter jejuni subsp. jejuni IA3902	100%	0.0	592	100.00%	P-type conjugative transfer protein
		Campylobacter coli RM1875	100%	0.0	578	91.82%	P-type conjugative transfer protein
		Campylobacter jejuni X	100%	0.0	577	91.51%	P-type conjugative transfer protein
		Campylobacter coli 132-6	100%	0.0	576	91.19%	P-type conjugative transfer protein
		Campylobacter sp. B423b	100%	0.0	575	91.19%	P-type conjugative transfer protein
**48**	WP_011799393.1	*Campylobacter jejuni subsp. jejuni IA3902*	100%	0.0	536	100.00%	TrbM-like protein
		Campylobacter jejuni subsp. jejuni 81-176	100%	0.0	536	99.62%	TrbM-like protein
		Campylobacter coli	100%	0.0	535	99.62%	TrbM-like protein
**49**	WP_011117588.1	*Campylobacter jejuni subsp. jejuni 81-176*	100%	7.00E-88	261	100.00%	toxin-antitoxin system protein
		Helicobacter canis NCTC 12740	71%	5.00E-61	191	100.00%	toxin-antitoxin system protein

S6 Table. Result of essential protein prediction using Geptop.

Sl no	Protein ID	Score
**1**	WP_002854524.1	1
**2**	WP_002854991.1	0.6004
**3**	WP_002855029.1	1
**4**	WP_002868905.1	0.9991
**5**	WP_002856929.1	0.4441
**6**	WP_011812736.1	1
**7**	WP_002869368.1	0.7991
**8**	WP_009882583.1	0.3995
**9**	WP_002853389.1	1
**10**	WP_009882608.1	0.799
**11**	WP_002856369.1	0.4539
**12**	WP_002831611.1	0.3632
**13**	WP_002853792.1	0.4539
**14**	WP_002869072.1	1
**15**	WP_002869097.1	0.4539
**16**	WP_002869326.1	0.2739
**17**	WP_002869139.1	0.3632
**18**	WP_002856630.1	0.3632
**19**	WP_002855458.1	0.285
**20**	WP_002797496.1	0.285
**21**	WP_024088174.1	0.4274
**22**	WP_002824979.1	0.4291
**23**	WP_002856602.1	0.5699
**24**	WP_002868888.1	1
**25**	WP_002868880.1	0.285
**26**	WP_002860117.1	0.5716
**27**	WP_002779704.1	0.4274
**28**	WP_011187233.1	0.5699
**29**	WP_011187235.1	0.5698
**30**	WP_002809111.1	1
**31**	WP_011117575.1	1
**32**	WP_011799393.1	1

S7 Table. List of predicted physicochemical parameters, sub-cellular localization for the HPs from C. jejuni.

										**Sub-cellular localization**		**Signal Peptide (Signal P)**	**Trans membrane helices prediction**		
**No**	Protein IDs	No of Amino acid	MW	PI	Extinction coefficient	Instability Index	Classification	Alphabatic index	Grand average of Hydropathicity (GRAVY)	**CELLO**	**PSORT B**		**HMMTOP**	**TMHMM**	**SOSUI**
**1**	WP_002868767.1	400	43408	9.11	22350	26.24	Stable	87.52	-0.337	OuterMembrane	OuterMembrane	YES	No	No	Membrane, 1 TM helix
**2**	WP_002854524.1	140	16278.72	4.75	19285	29.26	Stable	93.43	-0.209	Cytoplasmic	Cytoplasmic	No	No	No	Soluble
**3**	WP_009882162.1	241	26833.04	4.9	11920	35.93	Stable	43.9	-1.31	Extracellular	Unknown	No	No	No	Soluble
**4**	WP_010790856.1	136	15792.47	8.88	9190	28.8	Stable	77.5	-0.201	Cytoplasmic	Unknown	No	No	No	Soluble
**5**	WP_009882239.1	553	58834.74	4.79	33935	19.93	Stable	82.19	-0.41	Extracellular	OuterMembrane	YES	No	No	Soluble
**6**	WP_002854991.1	129	14888.85	5.31	11460	22.6	Stable	123.88	0.924	InnerMembrane	CytoplasmicMembrane	No	3 TM Helices	3 TM Helices	Membrane, 3 TM helix
**7**	WP_002855029.1	178	20660.76	4.93	34045	53.4	Unstable	101.4	-0.07	Cytoplasmic	Unknown	No	No	No	Soluble
**8**	WP_002868905.1	392	45037.15	9.63	52830	28.08	Stable	96.33	-0.377	OuterMembrane	Unknown	YES	No	No	Membrane, 1 TM helix
**9**	WP_002869356.1	360	41483.57	7.68	17880	31.74	Stable	98.06	-0.489	OuterMembrane	Cytoplasmic	No	1 TM Helices	1 TM Helices	Membrane, 1 TM helix
**10**	WP_002856929.1	238	27739.85	5.6	19160	40.81	Unstable	90.5	-0.789	Cytoplasmic	Cytoplasmic	No	No	No	Soluble
**11**	WP_002869028.1	441	51113.83	6.5	41385	23.72	Stable	70.27	-0.744	Cytoplasmic	Cytoplasmic	No	No	No	Soluble
**12**	WP_011812736.1	292	35243.21	9.33	29590	30.81	Stable	100.82	-0.395	Cytoplasmic	Cytoplasmic	No	No	No	Soluble
**13**	WP_002868809.1	408	46384.58	5.39	54125	24.69	Stable	92.89	-0.256	OuterMembrane	Unknown	YES	No	No	Soluble
**14**	WP_002869368.1	150	16506.86	6.58	7615	27.75	Stable	64.33	-0.486	Cytoplasmic	OuterMembrane	No	No	No	Soluble
**15**	WP_0098825	448	52423.	9.2	66365	36.19	Stable	85.8	-0.439	OuterMemb	Unknown	No	No	No	Membra

	83.1		83	7						rane					ne, 1 TM helix
**16**	WP_0028533 89.1	272	31893. 87	9.0 1	17545	42.12	Unstable	93.93	-0.638	Cytoplasmic	Cytoplasmic	No	No	No	Soluble
**17**	WP_0098826 08.1	372	42214. 61	4.8	20985	31.35	Stable	91.29	-0.388	Extracellular	OuterMembrane	No	No	No	Soluble
**18**	WP_0028563 69.1	138	15935. 31	5.0 3	9970	38.87	Stable	111.67	-0.227	Cytoplasmic	Unknown	YES	1 TM Helices	No	Soluble
**19**	WP_0792541 90.1	57	7067.7 8	10. 39	4470	32.55	Stable	114.56	0.053	Cytoplasmic	Unknown	YES	1 TM Helices	No	Membra ne, 1 TM helix
**20**	WP_0028561 80.1	64	7495.5 8	4.4 9	125	23.56	Stable	100.31	-0.302	Cytoplasmic	Unknown	No	No	No	Soluble
**21**	WP_0028316 11.1	87	10262. 55	6.0 7	18700	51.2	Unstable	63.79	-0.849	Cytoplasmic	Unknown	No	No	No	Soluble
**22**	WP_0027900 76.1	459	51016. 24	5.2 7	13535	43.38	Unstable	107.52	-0.068	OuterMemb rane	CytoplasmicMem brane	No	2 TM Helices	1 TM Helice s	Membra ne, 2 TM helix
**23**	WP_0028537 92.1	241	27299. 29	5.1 2	19495	28.46	Stable	102.66	0.028	Cytoplasmic	Unknown	YES	No	No	Membra ne, 1 TM helix
**24**	WP_0028690 72.1	316	37232. 84	6.2 3	19160	24.49	Stable	83.35	-0.187	Cytoplasmic	Cytoplasmic	No	No	No	Soluble
**25**	WP_0028690 97.1	452	52285. 74	9.4 2	36010	35.76	Stable	93.89	-0.373	Cytoplasmic	Cytoplasmic	No	No	No	Soluble
**26**	WP_0028693 26.1	464	52461. 9	7.1 1	39310	27.14	Stable	93.73	-0.289	OuterMemb rane	Unknown	No	1 TM Helices	No	Membra ne, 1 TM helix
**27**	WP_0028691 39.1	779	90202. 39	5.7 6	75360	43.31	Unstable	97.56	-0.315	Cytoplasmic	Cytoplasmic	No	No	No	Soluble
**28**	WP_0028691 95.1	65	7142.9 6	7.9 8	2980	6.5	Stable	75.23	-0.806	Periplasmic	Unknown	No	No	No	Soluble
**29**	WP_0028566 30.1	788	93550. 24	5.5 2	72020	36.88	Stable	93.68	-0.394	Cytoplasmic	Unknown	No	No	No	Soluble
**30**	WP_0028554 58.1	172	19575. 46	5.3 1	4470	37.6	Stable	96.51	-0.472	Cytoplasmic	Cytoplasmic	No	1 TM Helices	No	Membra ne, 1 TM helix
**31**	WP_0027974 96.1	142	16563. 95	9.0 1	7450	40.15	Unstable	75.63	-0.985	Cytoplasmic	Cytoplasmic	No	1 TM Helices	No	Soluble
**32**	WP_0240881 74.1	237	27971. 16	5.4 2	9190	49.19	Unstable	89.7	-0.393	Cytoplasmic	Cytoplasmic	No	No	No	Soluble
**33**	WP_0098830 30.1	439	52369. 05	5.6 6	48835	41.54	Unstable	103.87	-0.486	Cytoplasmic	Cytoplasmic	No	No	No	Soluble

**34**	WP_002824979.1	75	8773.25	6.27	5500	23.41	Stable	105.07	-0.167	Cytoplasmic	Unknown	No	No	No	Soluble
**35**	WP_002869225.1	288	32725.77	7.1	59610	30.26	Stable	113.75	0.844	InnerMembrane	CytoplasmicMembrane	No	8 TM Helices	8 TM Helices	Membrane, 8 TM helix
**36**	WP_002856602.1	138	15376.93	7.83	11460	17.73	Stable	98.91	-0.201	Periplasmic	Unknown	YES	No	No	Soluble
**37**	WP_002868888.1	328	39113.6	6.23	25370	26.75	Stable	95.43	-0.159	Cytoplasmic	Cytoplasmic	No	2 TM Helices	1 TM Helices	Membrane, 1 TM helix
**38**	WP_002868880.1	199	23051.39	8.95	24535	44.54	Unstable	101.01	-0.158	Extracellular	Unknown	No	2 TM Helices	No	Membrane, 1 TM helix
**39**	WP_009883121.1	166	18730.48	4.73	6085	23.14	Stable	99.94	-0.131	Cytoplasmic	Unknown	No	No	No	Soluble
**40**	WP_002860117.1	286	32643.66	5.19	37360	28.98	Stable	103.78	-0.095	Cytoplasmic	Cytoplasmic	No	No	No	Soluble
**41**	WP_002779704.1	88	10623.3	7.53	17795	57.44	Unstable	81.93	-0.601	Cytoplasmic	Unknown	No	No	No	Soluble
**42**	WP_011187233.1	408	47059.95	9.1	48025	28.47	Stable	79.39	-0.746	Cytoplasmic	Cytoplasmic	No	No	No	Soluble
**43**	WP_011187235.1	597	69031.68	7.94	51020	29.19	Stable	91.57	-0.431	OuterMembrane	Cytoplasmic	No	No	No	Soluble
**44**	WP_002809111.1	254	29365.92	8.88	39475	41.3	Unstable	79.84	-0.534	Periplasmic	Cytoplasmic	YES	No	No	Soluble
**45**	WP_011117548.1	225	25915.78	7.78	18910	24.92	Stable	93.16	-0.18	OuterMembrane	Unknown	No	2 TM Helices	1 TM Helices	Membrane, 1 TM helix
**46**	WP_011117549.1	356	40874.42	8.83	32320	34.91	Stable	80.06	-0.613	OuterMembrane	Unknown	YES	No	No	Soluble
**47**	WP_011117575.1	292	33294.59	5.67	19495	34.99	Stable	69.18	-0.692	Periplasmic	Unknown	YES	1 TM Helices	1 TM Helices	Soluble
**48**	WP_011799393.1	260	30029.93	8.54	60445	30.85	Stable	70.15	-0.637	OuterMembrane	Unknown	YES	1 TM Helices	No	Soluble
**49**	WP_011117588.1	134	15715.47	9.82	16960	38.64	Stable	90.82	-0.587	Cytoplasmic	Unknown	No	1 TM Helices	1 TM Helices	Soluble

Table S8. PPI of the predicted proteins from C. jejuni

SL	Protein ID	Interacted protein	Score
**1**	WP_002868767.1	Lipoprotein, putative (207 aa)	0.945
**2**	WP_002854524.1	Flagellar motor switch protein FliN (102 aa)	0.87
**3**	WP_009882162.1	HIT family protein (120 aa)	0.634
**4**	WP_010790856.1	Pyridoxine 5'-phosphate synthase	0.9
**5**	WP_009882239.1	Putative outer-membrane protein (508 aa)	0.908
**6**	WP_002854991.1	Porphobilinogen deaminase (EC-2.5.1.61)	0.861
**7**	WP_002855029.1	DNA polymerase III, delta prime subunit, homolog (199 aa)	0.964
**8**	WP_002868905.1	Uncharacterized protein (336 aa)	0.973
**9**	WP_002869356.1	Ketol-acid reductoisomerase (EC-1.1.1.86) (340 aa)	0.882
**10**	WP_002856929.1	No result	NA
**11**	WP_002869028.1	ulfate-binding protein precursor (348 aa)	0.884
**12**	WP_011812736.1	Sensor protein ZraS (EC-2.7.13.3) (339 aa)	0.859
**13**	WP_002868809.1	NADP-dependent 3-hydroxy acid dehydrogenase YdfG (EC-1.1.1.-) (249 aa)	0.888
**14**	WP_002869368.1	hermonuclease precursor (EC-3.1.31.1) (175 aa)	0.533
**15**	WP_009882583.1	ATP-dependent RecD-like DNA helicase (EC-3.6.4.12) (447 aa)	0.674
**16**	WP_002853389.1	Membrane protein insertase YidC	0.889
**17**	WP_009882608.1	ABC transporter glutamine-binding protein GlnH precursor (279 aa)	0.643
**18**	WP_002856369.1	Rhomboid protease AarA (EC-3.4.21.105) (157 aa)	0.614
**19**	WP_079254190.1	No result	NA
**20**	WP_002856180.1	Cadmium, cobalt and zinc/H(+)-K(+) antiporter (316 aa)	0.875
**21**	WP_002831611.1	Uncharacterized protein (64 aa)	0.681
**22**	WP_002790076.1	Chemotaxis protein CheA (EC-2.7.13.3) (769 aa)	0.978
**23**	WP_002853792.1	Plasminogen-binding protein PgbB (332 aa)	0.904
**24**	WP_002869072.1	Murein DD-endopeptidase MepM (EC-3.4.24.-) (273 aa)	0.859
**25**	WP_002869097.1	3-oxoacyl-[acyl-carrier-protein] synthase 3 (EC-2.3.1.180) (353 aa)	0.86
**26**	WP_002869326.1	1-deoxy-D-xylulose 5-phosphate reductoisomerase (EC-1.1.1.267)	0.862
**27**	WP_002869139.1	Pyruvate-flavodoxin oxidoreductase (EC-1.2.7.-)	0.979
**28**	WP_002869195.1	Uncharacterized protein (144 aa)	0.712
**29**	WP_002856630.1	ATP-dependent helicase/nuclease subunit A (EC-3.1.-.-) (921 aa)	0.991
**30**	WP_002855458.1	Adenylosuccinate synthetase (EC-6.3.4.4)	0.883
**31**	WP_002797496.1	Adenylosuccinate synthetase	0.911
**32**	WP_024088174.1	Formate dehydrogenase	0.893
**33**	WP_009883030.1	Uncharacterized protein (282 aa)	0.859
**34**	WP_002824979.1	NADH dehydrogenase subunit C	0.987
**35**	WP_002869225.1	Anaerobic dimethyl sulfoxide reductase chain A (774 aa)	0.956
**36**	WP_002856602.1	Uncharacterized protein (247 aa)	0.754
**37**	WP_002868888.1	Uncharacterized protein (183 aa)	0.88

**38**	WP_002868880.1	ABC transporter, periplasmic substrate-binding protein, putative (296 aa)	0.867
**39**	WP_009883121.1	Lipoprotein, putative (199 aa)	0.676
**40**	WP_002860117.1	Aminodeoxyfutalosine synthase (EC-2.5.1.-)	0.988
**41**	WP_002779704.1	No result	NA
**42**	WP_011187233.1	No result	NA
**43**	WP_011187235.1	No result	NA
**44**	WP_002809111.1	No result	NA
**45**	WP_011117548.1	No result	NA
**46**	WP_011117549.1	VirB10	0.994
**47**	WP_011117575.1	No result	NA
**48**	WP_011799393.1	No result	NA
**49**	WP_011117588.1	No result	NA

Table S9. 3D structural information of HPs from C. Jejuni

SL No	Accession No	Templates	Domain and function in (PS)2-v2	% Identity	Organism	Method	Resolution	R-value free	R-value work
1	WP_002868767.1	1efcA	PROTEIN (ELONGATION FACTOR)	51.58%	*Escherichia coli*	X-RAY DIFFRACTION	2.05 Å	0.268	0.203
2	WP_002854524.1	1squB	Chemotaxis phosphatase CheX	71.52%	*Thermotoga maritima*	X-RAY DIFFRACTION	2.4 Å	0.320	0.239
3	WP_009882162.1	No results							
4	WP_010790856.1	2ig6A	NimC/NimA family protein	87.88%	*Clostridium acetobutylicum*	X-RAY DIFFRACTION	1.8 Å	0.198	0.168
5	WP_009882239.1	No results							
6	WP_002854991.1	1oedB	ACETYLCHOLINE RECEPTOR PROTEIN, ALPHA CHAIN	89.19%	*Torpedo marmorata*	ELECTRON MICROSCOPY	4 Å		
7	WP_002855029.1	2uvpC	DNA replication regulator HOBA	68.48%	*Helicobacter pylori*	X-RAY DIFFRACTION	1.7 Å	0.214	0.180
8	WP_002868905.1	1yzfA	lipase/acylhydrolase	79.01%	*Enterococcus faecalis*	X-RAY DIFFRACTION	1.9 Å	0.239	0.184
9	WP_002869356.1	No results							
10	WP_002856929.1	No results							
11	WP_002869028.1	1l3wA	EP-cadherin	62.14%	*Xenopus laevis*	X-RAY DIFFRACTION	3.08 Å	0.276	0.243
12	WP_011812736.1	2e52A	Type II restriction enzyme HindIII	71.07%	*Haemophilus influenzae*	X-RAY DIFFRACTION	2 Å	0.217	0.175
13	WP_002868809.1	1n11A	Ankyrin	77.69%	*Homo sapiens*	X-RAY DIFFRACTION	2.7 Å	0.303	0.319
14	WP_002869368.1	1eemA	GLUTATHIONE-S-TRANSFERASE	67.95%	*Homo sapiens*	X-RAY DIFFRACTION	2 Å	0.271	0.219
15	WP_009882583.1	No results							
16	WP_002853389.1	No results							
17	WP_009882608.1	2c5uA	RNA LIGASE	54.57%	*Enterobacteria phage T4*	X-RAY DIFFRACTION	2.21 Å	0.258	0.198
18	WP_002856369.1	3dueA	Putative periplasmic protein	88.80%	*Bacteroides vulgatus*	X-RAY DIFFRACTION	1.85 Å	0.233	0.192
19	WP_079254190.1	No result							
20	WP_002856180.1	1osdA	hypothetical protein MerP	70.49%	*Cupriavidus metallidurans*	X-RAY DIFFRACTION	2 Å	0.268	0.192
21	WP_002831611.1	2a2pA	Selenoprotein M	64.71%	*Mus musculus*	SOLUTION NMR			
22	WP_002790076.1	2ch7A	METHYL-ACCEPTING	89.94%	*Thermotoga maritima*	X-RAY DIFFRACTION	2.5 Å	0.297	0.259

			CHEMOTAXIS PROTEIN						
23	WP_002853792.1	No result							
24	WP_002869072.1	1zkdA	DUF185	73.10%	*Rhodopseudomonas palustris*	X-RAY DIFFRACTION	2.1 Å	0.258	0.223
25	WP_002869097.1	No results							
26	WP_002869326.1	No results							
27	WP_002869139.1	No results							
28	WP_002869195.1	No result							
29	WP_002856630.1	1w36F	EXODEOXYRIBONUCLEASE V BETA CHAIN	62.78%	*Escherichia coli*	X-RAY DIFFRACTION	3.1 Å	0.296	0.242
30	WP_002855458.1	1c1gA	TROPOMYOSIN	86.59%	*Sus scrofa*	X-RAY DIFFRACTION	7 Å		0.404
31	WP_002797496.1	2efrA	General control protein GCN4 and Tropomyosin 1 alpha chain	91.67%	*Saccharomyces cerevisiae , Oryctolagus cuniculus*	X-RAY DIFFRACTION	1.8 Å	0.316	0.237
32	WP_024088174.1	1n1cA	TorA specific chaperone	84.46%	*Shewanella massilia*	X-RAY DIFFRACTION	2.4 Å	0.255	0.224
33	WP_009883030.1	No results							
34	WP_002824979.1	1r8sA	ADP-ribosylation factor 1	77.78%	*Bos taurus, Homo sapiens*	X-RAY DIFFRACTION	1.46 Å	0.170	0.159
35	WP_002869225.1	2dyrA	Cytochrome c oxidase subunit 1	80.54%	*Bos taurus*	X-RAY DIFFRACTION	1.8 Å	0.227	0.202
36	WP_002856602.1	3db7A	putative calcium-regulated periplasmic protein	88.00%	*Bacteroides thetaiotaomicron*	X-RAY DIFFRACTION	1.4 Å	0.198	0.160
37	WP_002868888.1	No results							
38	WP_002868880.1	2iqiF	Hypothetical protein XCC0632	82.94%	*Xanthomonas campestris*	X-RAY DIFFRACTION	2.7 Å	0.275	0.209
39	WP_009883121.1	1quuA	HUMAN SKELETAL MUSCLE ALPHA-ACTININ 2	76.51%	*Homo sapiens*	X-RAY DIFFRACTION	2.5 Å	0.310	0.229
40	WP_002860117.1	1zbmA	hypothetical protein AF1704	79.92%	*Archaeoglobus fulgidus*	X-RAY DIFFRACTION	2.3 Å	0.265	0.212
41	WP_002779704.1	2cm5A	RABPHILIN-3A	72.22%	*Rattus norvegicus*	X-RAY DIFFRACTION	1.28 Å	0.194	
42	WP_011187233.1	2au3A	DNA primase	58.81%	*Aquifex aeolicus*	X-RAY DIFFRACTION	2 Å	0.238	0.203
43	WP_011187235.1	No results							
44	WP_002809111.1	3ec1A	YqeH GTPase	53.75%	*Geobacillus stearothermophilus*	X-RAY DIFFRACTION	2.36 Å	0.287	0.254
45	WP_011117548.1	No results							
46	WP_011117549.1	2ofqA	TraO	79.38%	*Salmonella*	SOLUTION NMR			

					typhimurium				
47	WP_011117575.1	2ch7A	METHYL-ACCEPTING CHEMOTAXIS PROTEIN	81.99%	*Thermotoga maritima*	X-RAY DIFFRACTION	2.5 Å	0.297	0.259
48	WP_011799393.1	1zsoB	hypothetical protein	73.97%	*Plasmodium falciparum*	X-RAY DIFFRACTION	2.17 Å	0.233	0.183
49	WP_011117588.1	1ne8A	conserved hypothetical protein YDCE	56.56%	*Bacillus subtilis*	X-RAY DIFFRACTION	2.1 Å	0.210	0.159
